# Short metallopeptide conjugate nanostructures for selective cadmium capture and detoxification

**DOI:** 10.1039/d5na00969c

**Published:** 2025-12-03

**Authors:** Aanand Kautu, Shruti Sharma, Nikunj Kumar, Ashwini Waghmare, Bodhisattwa Das Gupta, Sudipta Mondal, Puneet Gupta, Yogesh Bhargava, Khashti Ballabh Joshi

**Affiliations:** a Department of Chemistry, School of Chemical Science and Technology, Dr Harisingh Gour Vishwavidyalaya (A Central University) Sagar MP 470003 India kbjoshi77@gmail.com kbjoshi@dhsgsu.ac.in; b Computational Catalysis Centre, Department of Chemistry, Indian Institute of Technology Roorkee 247667 Uttarakhand India; c Molecular Engineering and Imaging Lab, Department of Microbiology, Dr Harisingh Gour Vishwavidyalaya (A Central University) Sagar M. P. 470003 India yogesh.bhargava@gmail.com; d Department of Biotechnology, National Institute of Technology Durgapur-713209 West Bengal India

## Abstract

We report a rationally designed biomimetic supramolecular short metallopeptide conjugate (sMPC) that unites sensitive cadmium detection with active detoxification. These cost-effective and biocompatible assemblies respond to Cd^2+^ by precise metal chelation and stimuli-induced self-assembly, producing a marked fluorescence enhancement for quantification while driving a controlled morphological conversion from cadmium nanoparticles to less toxic nanorods and stabilizing key intermediates. Integrated spectroscopic, microscopic and theoretical analyses reveal the molecular mechanisms of this dynamic organization, advancing fundamental understanding of peptide–metal interactions. Proof-of-concept *in vitro* (HEK-293) and *in vivo*-zebrafish-study assays, demonstrate efficient cadmium clearance, mitigation of oxidative stress and cellular recovery. By combining real-time sensing with a built-in detoxification pathway, this human- and environment-friendly nanoarchitectonic platform provides a transformative strategy for heavy-metal toxicity mitigation and opens avenues for next-generation biomaterials in environmental monitoring and intracellular metal detoxification.

## Abstract 2

A biomimetic metallopeptide conjugate enables stimuli-responsive cadmium capture and detoxification, offering a novel platform for environmental monitoring and therapeutic chelation.

## Graphical abstract

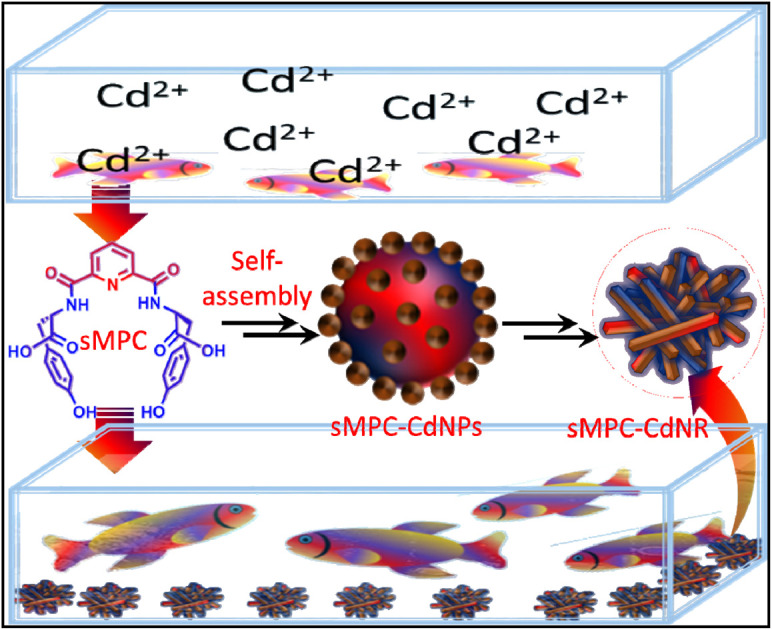

Cadmium (Cd) is a naturally occurring metal in the Earth's crust, yet it has no beneficial role in the human body.^1,2^ Instead, it is highly toxic^3^ and an escalating concern for environmental scientists and medical researchers alike. Its extensive industrial use-in batteries, pigments, coatings, and other products-means Cd often leaks into places it should never be: our food, water, and air.^4^ Water is the main route for its spread; Cd dissolves readily, binds to other substances, and persists without degradation, accumulating in plants and animals and posing a long-term threat to ecosystems and health.^1,5^ Aquatic organisms are among the first to signal contamination, acting as living indicators of pollution.^1,5^ To anticipate Cd's impact on human health, researchers frequently turn to zebrafish-small but biologically powerful models^4^-which display stress responses, developmental abnormalities, and physiological damage upon Cd exposure, offering valuable clues for protective strategies.^4^

In humans, cadmium (Cd) is not readily excreted; instead, it accumulates in critical organs such as the kidneys, liver, lungs, bones and brain, where it can persist for decades.^6^ Chronic retention disrupts normal physiology by generating reactive oxygen species (ROS) that damage lipids, proteins and nucleic acids, undermining cellular integrity.^6,7^ Cadmium also impairs DNA repair pathways and mitochondrial energy production, weakening natural defence mechanisms and promoting long-term tissue injury.^6,7^ This cumulative toxicity manifests as renal dysfunction, bone demineralisation, neurotoxicity and immune suppression, placing vulnerable populations at heightened risk. Understanding these molecular and systemic effects of Cd exposure is therefore a crucial step toward developing strategies to protect human health and safeguard the environment.^8^

Cadmium detoxification poses a persistent biomedical challenge, largely because the body's intrinsic defence systems respond to Cd^2+^ in complex and sometimes counterproductive ways.^8,9^ Metallothioneins, for example, sequester cadmium ions and can transiently reduce their free concentration, yet this same binding may prolong the metal's residence time within tissues.^10^ Conversely, depletion of glutathione-one of the cell's principal antioxidants-amplifies oxidative stress, disrupts redox balance and accelerates cell injury.^11^ Unfortunately, existing chelation therapies offer little relief for cadmium toxicity (Tables S1 and S2): widely used agents such as EDTA, DMSA and BAL show limited efficacy against tissue-bound Cd and can induce adverse effects, including nausea, anorexia and even depletion of essential metal ions.^9,12^ This combination of intricate endogenous responses and inadequate pharmacological tools underscores the urgent need for more cost effective, biocompatible strategies for cadmium detoxification.

Heavy-metal toxicity, particularly from cadmium (Cd^2+^), represents a severe and persistent threat to environmental and human health, yet existing detoxification strategies lack the requisite selectivity, biocompatibility and real-time monitoring capabilities.^9,12,13^ In response, we have engineered a new class of short metallopeptide conjugates (sMPCs), exemplified by pyridine-*bis*-tyrosine (Fig. 1)^13,14^, using a minimalist design that integrates nanotechnology with peptide-based materials.^13,15,16^ This sMPC comprises tyrosine residues linked *via* a rigid, C_2_-symmetric pyridine core, generating crescent-shaped architectures that selectively bind toxic metal ions (Fig. 2, 3 and S1).^13^ Their stimuli-responsive self-assembly and intrinsic optoelectronic properties confer dual functions-detoxification and detection.^17^ Recent advances in noncovalent peptide glasses have revealed how short peptides can form highly dynamic, amorphous nanoarchitectures through LLPS-mediated assembly.^18,19^ These studies established short peptides as versatile platforms for adaptive nanostructuring. Building on this paradigm, our work extends the concept to metal-responsive systems, showing that a rationally designed metallopeptide can sense Cd^2+^ and undergo controlled structural transitions that directly couple detection with detoxification.

**Fig. 1:**
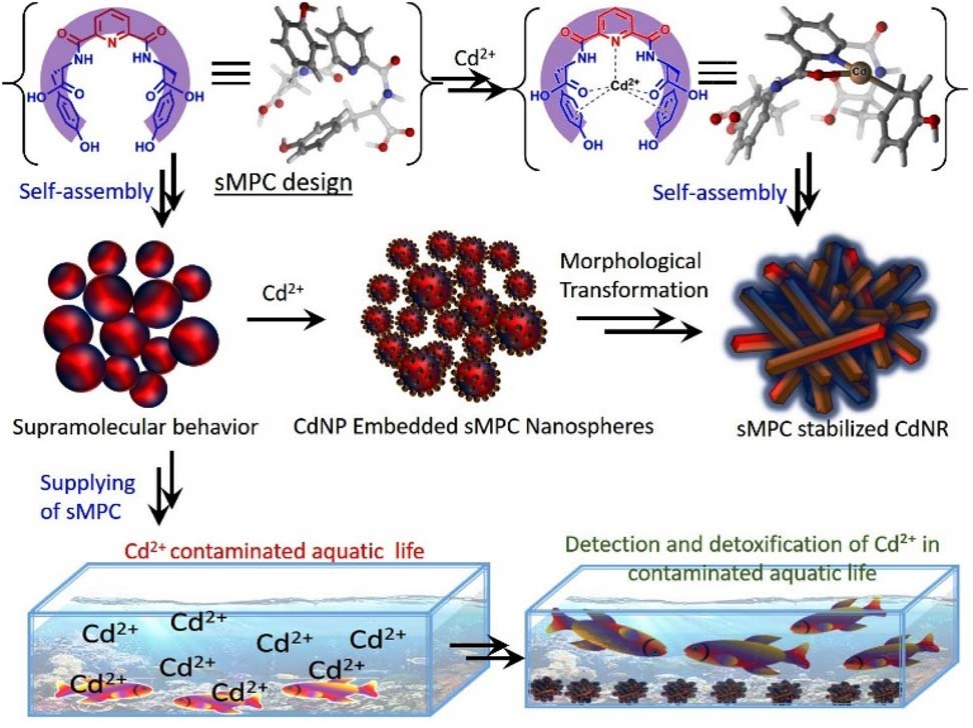
The crescent-shaped sMPC, a pyridine-*bis*-tyrosine conjugate, coordinates Cd^2+^ by minimizing chain fluctuations, enabling spherical self-assembly. This interaction induces Cd^2+^ nanoparticles (CdNPs) formation, maturing into nanorods (CdNRs). sMPC scavenges Cd^2+^, offering eco-bioremedial potential in aquatic and biological systems.

**Fig. 2:**
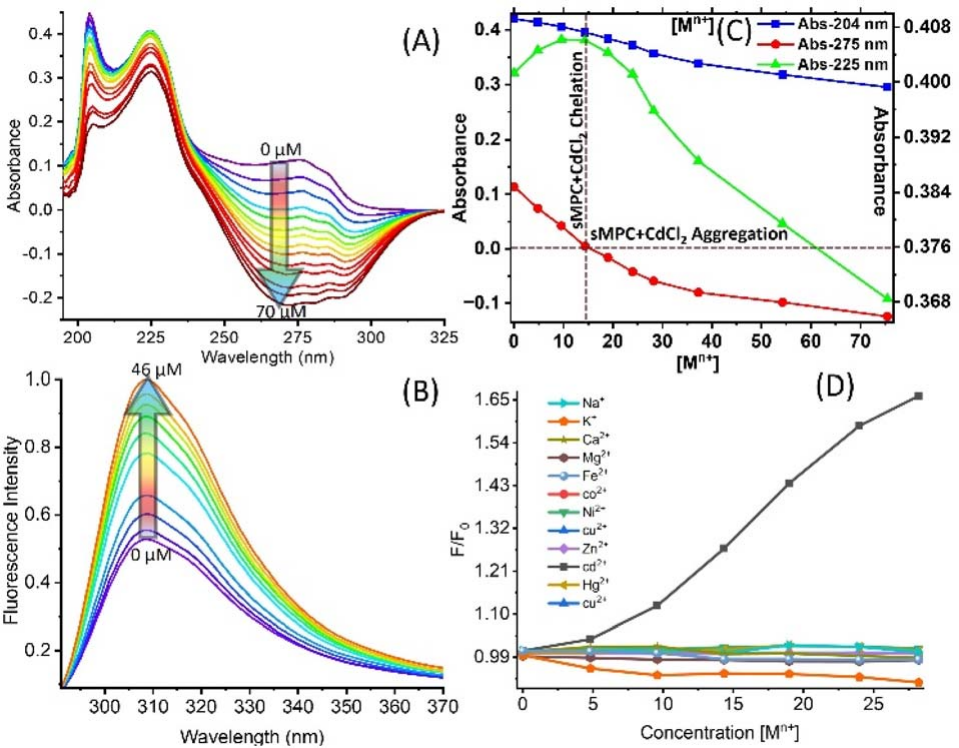
Depicts: (A) UV-Vis spectra show a concentration-dependent decrease at 275 nm, indicating tyrosine π–π* transition perturbation due to Cd^2+^ coordination or intermolecular interactions. (B) Fluorescence spectra (*λ*_ex_ = 260 nm) exhibit enhanced emission at 310 nm *via* chelation-enhanced fluorescence (CHEF). (C) Job's plot using absorbance shifts at 204, 225, and 275 nm confirms sMPC–Cd^2+^ complex formation with aggregation. (D) *F*/*F*_0_*vs.* [M^n+^] titrations reveal a uniquely strong CHEF response for Cd^2+^, demonstrating the exceptional affinity and selective sensing capability of sMPCs toward cadmium, consistent with their relevance for Cd^2+^ detection and detoxification.

**Fig. 3:**
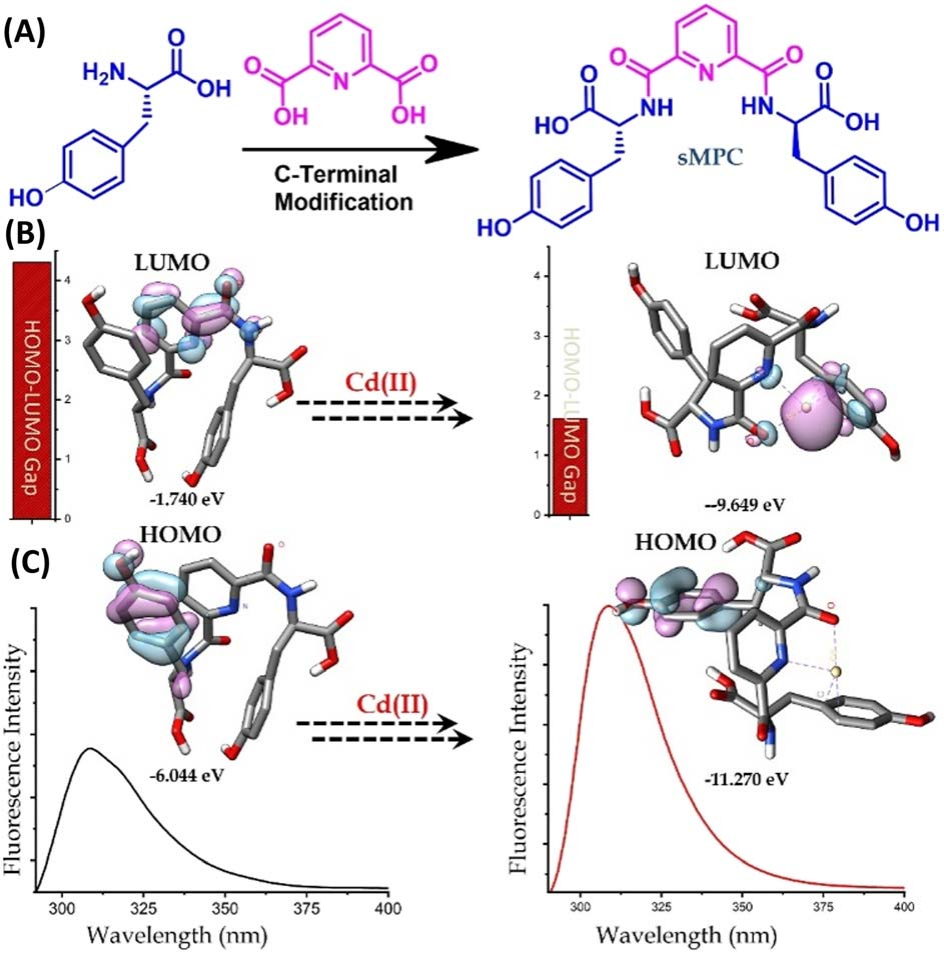
Design and Cd^2+^-responsive optical behaviour of sMPC. (A) Tyrosine residues conjugated to pyridine-2,6-dicarboxylic acid yield a C-terminally modified, crescent-like its *bis* conjugates (metallopeptide conjugate). (B and C) Frontier molecular orbital analysis shows marked changes in HOMO/LUMO localization and a narrowed energy gap upon Cd^2+^ binding, indicating favourable electronic rearrangement. Fluorescence spectra (bottom) display chelation-enhanced emission consistent with metal-induced structural reorganisation and orbital redistribution, corroborating the experimentally observed “turn-on” fluorescence effect.

Upon Cd^2+^ exposure, sMPCs display fluorescence enhancement (Fig. 2B) and adopt a more ordered conformation, confirmed by energy-minimized modelling, HOMO–LUMO analysis and DFT simulations^13,15,20,21^ (Fig. 3 and S1). Unlike free tyrosine (Fig. S2), which fails to form stable complexes, sMPCs create a defined metal-binding cavity stabilized by cation–π interactions and coordination through pyridinyl nitrogen and phenolic oxygen, with a calculated binding energy of −221.8 kcal mol^−1^ (Fig. S1). Experimental validation using UV, IR and fluorescence spectroscopy^22,23^ (Fig. 2 and 4) demonstrated robust Cd^2+^ chelation. Collectively, these findings support sMPCs as multifunctional platforms for real-time monitoring and safe detoxification of heavy metals,^13,20,24^ offering a promising route to address urgent environmental and biomedical challenges.

**Fig. 4:**
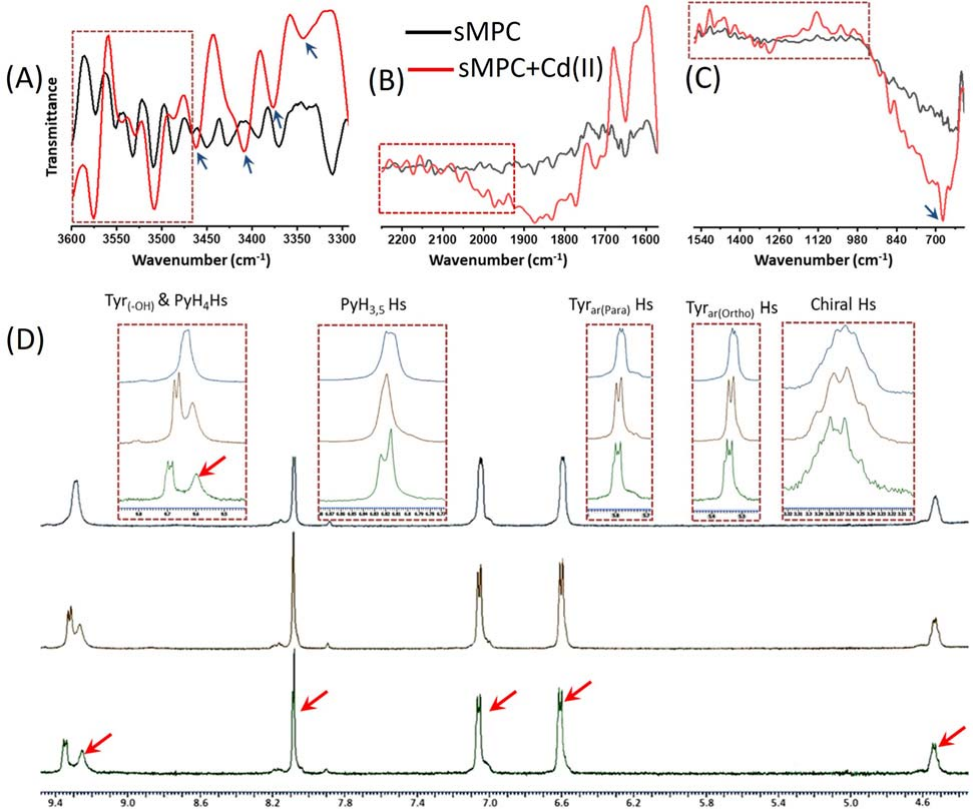
FT-IR (A–C) and ^1^H NMR (D) reveal molecular interactions between Cd^2+^ and sMPC. FT-IR (black *vs.* red) shows *ν*(OH/NH) shifting 3311 → 3343 cm^−1^, amide I/II perturbations, a new 676 cm^−1^ band (Cd–O/N) and changes near 1290 cm^−1^ (C–O/C–N) indicating direct coordination. ^1^H NMR titration exhibits PyH_3_,_5_ doublet → singlet, PyH_4_/Tyr aromatic triplet → doublet shifts and Tyr–OH loss with signal broadening. Together these data demonstrate Cd^2+^ binding *via* phenolic, amide and pyridyl N/O donors, informing sMPC sequestration strategies.

To elucidate the interaction of sMPC with Cd^2+^, UV-Vis spectroscopy revealed a progressive decrease in absorbance at 275 nm upon titration with Cd^2+^, indicative of chelation-induced conformational rearrangement and transition to more compact or aggregated states (Fig. 2A and B).^25–27^ This Cd^2+^-triggered aggregation correlates with a marked fluorescence “turn-on” effect, consistent with tyrosine quenching loss and enhanced emissive pathways. Job's plot confirmed stable sMPC-Cd^2+^complex stoichiometry, while Stern–Volmer analysis demonstrated high binding affinity and pronounced selectivity for Cd^2+^ (Fig. S5). Fluorescence intensity at 310 nm (*λ*_ex_ = 260 nm) increased linearly with Cd^2+^ concentration, validating specificity and quantification capability.^23,27^ The linear correlation observed between the fluorescence intensity ratio (*F*/*F*_0_) and Cd^2+^ concentration demonstrates the reliability and predictability of the sensing response across the tested concentration range. This strong linearity indicates that the system can be effectively applied for quantitative monitoring of Cd^2+^ levels with high reproducibility and accuracy (Fig. S5).^13^ To evaluate the metal-ion selectivity of sMPC, we examined its interaction with a comprehensive panel of biologically and environmentally relevant cations, including Na^+^, K^+^, Mg^2+^, Ca^2+^, Fe^2+^, Fe^3+^, Co^2+^, Ni^2+^, Zn^2+^, Cu^2+^, Cd^2+^, and Hg^2+^. With the exception of Cd^2+^ and Fe^3+^, all tested ions exhibited negligible or very weak interaction with sMPC. Although both Cd^2+^ and Fe^3+^ bind strongly at the spectroscopic level, their photophysical outcomes are markedly different: Cd^2+^ complexation affords the fluoresce enhancement and formation of CdNR species through a pronounced CHEF effect, whereas Fe^3+^ induces significant quenching (CHEQ), likely *via* paramagnetic or LMCT-mediated non-radiative pathways. Consistent with HSAB principles, the soft donor environment of sMPC preferentially stabilizes Cd^2+^ over Fe^3+^, leading to a more robust and structurally defined Cd–sMPC assembly. Under physiologically relevant zebrafish conditions and natural water matrices, the availability of free Fe^3+^ is extremely limited due to biological sequestration and rapid hydrolytic precipitation, while Cd^2+^ remains soluble and accessible. Consequently, despite detectable Fe^3+^ binding *in vitro*, sMPC predominantly captures Cd^2+^ under competitive conditions and efficiently forms CdNR nanostructures, enabling selective Cd^2+^ detoxification without disrupting iron homeostasis.

To probe Cd^2+^-induced structural and functional changes in sMPC, we combined ^1^H NMR, FT-IR, and cyclic voltammetry (CV) (Fig. 4 and S3). ^1^H NMR spectra showed marked perturbations upon Cd^2+^ binding: aromatic signals of the pyridine moiety (PyH_3_,_5_) collapsed into a singlet, PyH_4_ and tyrosine aromatic protons shifted from triplets to doublets, and the Tyr-OH resonance disappeared-signatures of strong, possibly dynamic coordination involving pyridinyl nitrogen, phenolic hydroxyl, and amine groups.^27,28^ FT-IR analysis corroborated these interactions, with O–H/N–H stretches shifting from 3311 to 3343 cm^−1^ and new fingerprint peaks emerging at 676 cm^−1^ (Cd–O/N stretching); additional shifts near 1290 cm^−1^ reflected altered C–O and C–N bonds, while amide I/II region changes confirmed backbone involvement.^13,14^ CV studies further revealed two tyrosine-related oxidation peaks at ∼0.5 V and 1.2 V (Fig. S3);^29,30^ upon Cd^2+^ addition these peaks diminished, and at higher Cd^2+^ levels vanished entirely, indicating that complex formation stabilizes electron-donating groups and hinders electron transfer *via* aggregation or steric effects. Together, these complementary spectroscopic and electrochemical results establish stable, multi-site Cd^2+^ coordination by sMPC and illuminate the electronic and redox changes underlying its high potential for cadmium sensing and detoxification.^29,30^ Cadmium ions induce a dynamic, time-resolved transformation in the self-assembly of sMPC (Fig. 5A) into sMPC–Cd nanostructures (Fig. 5B–D), as visualised by TEM. Initially, 250 µM sMPC solution forms uniform spherical nanostructures (Fig. 5A and 6A, B).^14^ Upon Cd^2+^ addition, ultrasmall Cd nanoparticles (CdNPs)^31,32^ nucleate within or decorate the peptide spheres (Fig. 5B and 6C, D), reflecting strong metal–ligand coordination with sMPC donor groups.^32–34^ Enhanced image contrast and internal granularity support *in situ* nucleation and chelation-mediated growth of CdNPs (Fig. 6E), with histogram analysis confirming a narrow size distribution (∼5.69 ± 0.01 nm, Fig. 6F).

**Fig. 5:**
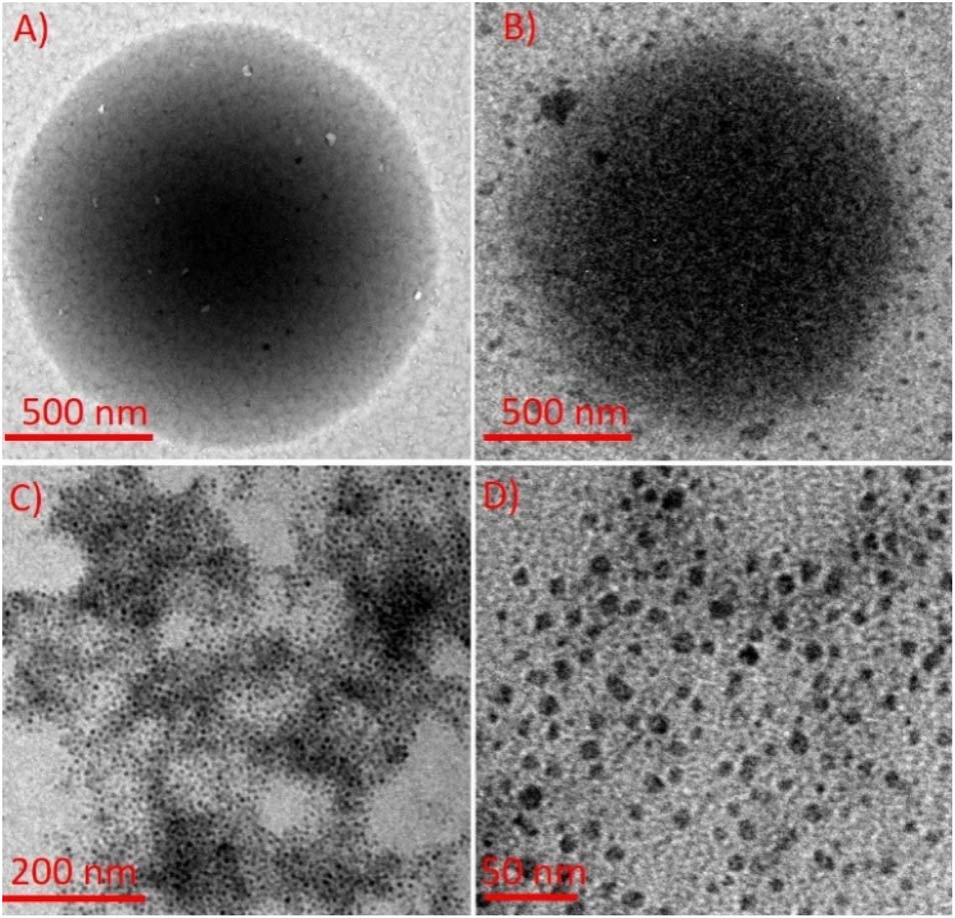
TEM images show (A) self-assembled sMPC nanostructures and (B) CdCl_2_-treated sMPC with embedded, uniformly distributed CdNPs indicating nucleation/stabilization. (C) High CdNP density on surfaces; (D) enlarged view suggests effective stabilization *via* sMPC coordination interactions.

**Fig. 6:**
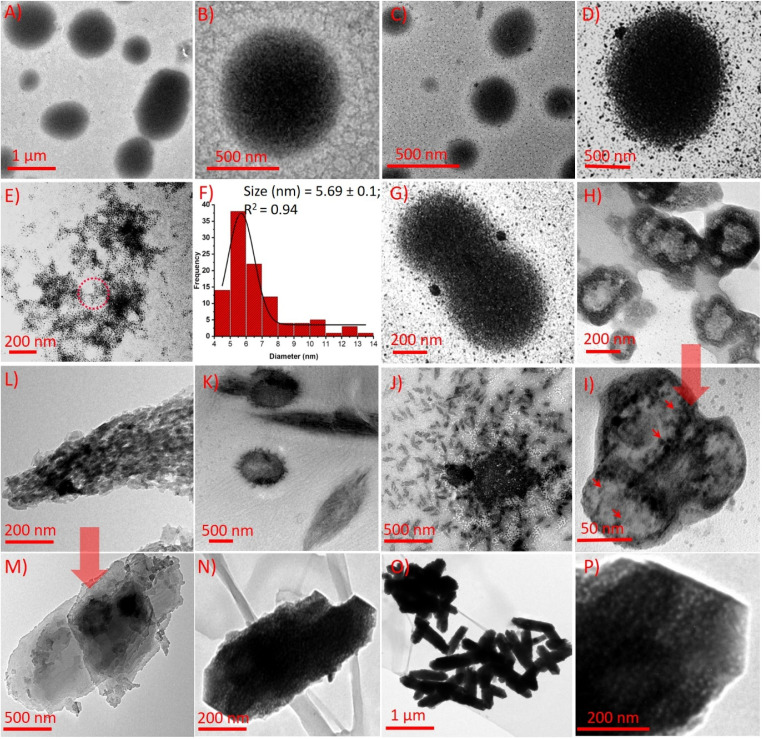
Formation of cadmium nanorods (CdNRs) from cadmium nanoparticles (CdNPs). TEM images show sMPCs (250 µM) forming uniform spherical nanostructures (A and B). Upon Cd^2+^ addition, *in situ* nucleation yields CdNPs embedded in the peptide matrix (C and D). Ultrasmall, monodispersed CdNPs are seen across the grid (E), with size distribution shown (F). Panels G–P depict time-dependent evolution from CdNPs to CdNRs. Fusion begins at 6 h (G), followed by distorted spheres *via* Ostwald ripening (H–I), elongation (J–L), and layered sheet formation (M), culminating in mature CdNRs by 48 h (N–P), highlighting hierarchical Cd^2+^-driven self-assembly within the metallopeptide scaffold.

Time-dependent TEM images reveal morphological evolution from CdNP-bearing spheres to anisotropic forms. At 6 h (Fig. 6G–I), sphere fusion signals onset of oriented growth; by 12–18 h distorted spheres emerge, consistent with Ostwald ripening^18^ (Fig. 3J and K). Between 24–36 h (Fig. 6L and M), intermediates transform into elongated, layered sheets, culminating at 48 h in well-defined Cd nanorods (CdNRs)^35–37^ (Fig. 6N and P). Magnified views (Fig. 6P) highlight porous, fused features suggesting linear attachment of CdNPs stabilised by sMPC. This cadmium-driven assembly enhances Cd^2+^ capture and, coupled with sMPC's biocompatibility and highlight porous, fused features suggesting linear attachment of CdNPs stabilised by sMPC. This cadmium-driven assembly enhances Cd^2+^ capture and, coupled with sMPC's biocompatibility and degradability, offers a safer alternative to EDTA and other chelants, paving the way for next-generation detoxification and therapeutic nanomaterials.^13–15,37,38^ A comprehensive literature review on cadmium-based nanoparticles highlights major advances yet clear gaps in structural diversity and real-time applicability (Tables S1 and S2). Our study addresses this by introducing a novel CdNPs/CdNRs-sMPC system. As detailed in Tables S1 and S2 (SI), its synthesis, structural characterisation and functional evaluation expand fundamental understanding and demonstrate practical, real-time potential, underscoring the originality and relevance of this work in advancing cadmium nanomaterials.

In line with recent advances in LLPS-mediated peptide self-assembly,^39–41^ our results suggest that the metallopeptide first undergoes liquid–liquid phase separation to form dynamic, solute-rich nanodroplets that serve as precursors for nucleation. These droplets gradually fuse through surface tension-driven coalescence, followed by internal molecular reorganization that increases local ordering. This hierarchical process ultimately triggers anisotropic nucleation and growth, giving rise to the observed nanofibrillar and rod-like architectures. Guided by these principles, we now present a unified mechanistic model (Fig. 7) that describes LLPS-driven droplet formation,^18^ sphere fusion, and subsequent structural maturation in our system, providing a coherent framework that rationalizes the dynamic transitions captured in our spectroscopic and microscopic analyses.

**Fig. 7:**
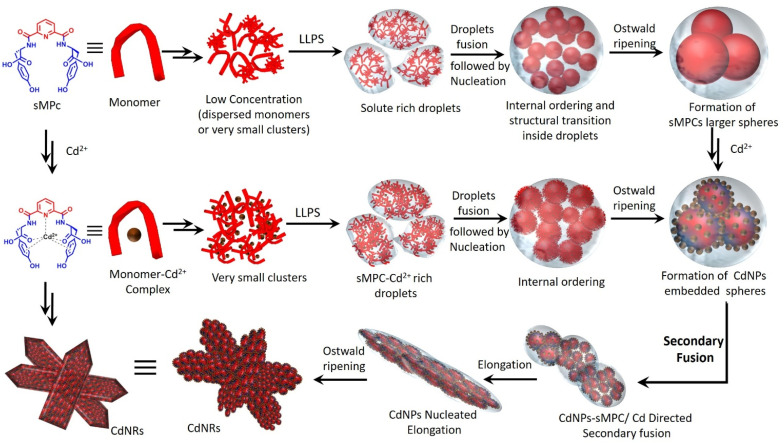
Proposed mechanistic model illustrating the formation of sMPC spheres *via* an LLPS-driven pathway, followed by their maturation into Cd-bound nanoparticles (CdNPs). Initial peptide–metal condensation droplets undergo LLPS, fusion, and internal reorganization, ultimately transitioning into ordered CdNRs assemblies.^18,19^

Transmission electron microscopy (TEM) images (Fig. 8) further demonstrate the successful formation of cadmium nanorods (CdNRs) stabilized by sMPC molecules. Panels left and right show a dense distribution of CdNRs, indicating efficient stabilization and growth under the applied conditions. A closer view in panel right magnified panel (Fig. 8) suggests that these nanorods likely evolve through a stepwise mechanism involving the linear fusion or oriented attachment of smaller cadmium nanoparticles (CdNPs), consistent with previously reported assembly pathways for anisotropic nanostructures. The inset highlights porous-like contrast features, which are most plausibly attributed to electron scattering variations rather than actual structural porosity. Panels right provide successive magnifications of the nanorods depicted in panel left, with the inset in (Fig. 8 right) clearly showing fused domains along the nanorod axis, thereby supporting the proposed nucleation, attachment, and growth mechanism facilitated by sMPC stabilization. Collectively, these observations suggest that sMPC not only prevents uncontrolled aggregation but also directs the anisotropic assembly of CdNPs into ordered CdNRs.

**Fig. 8:**
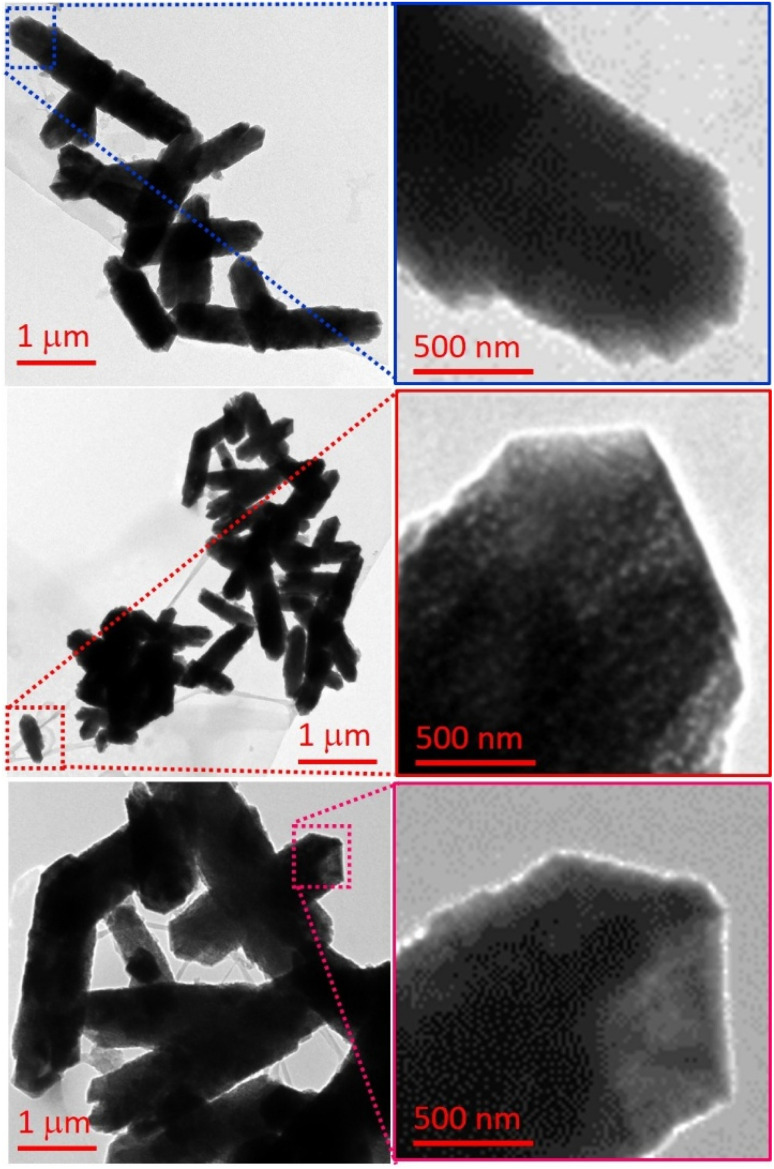
Different regions of TEM images showing cadmium nanorods (CdNRs) stabilized by sMPC. Panels (left–right) display abundant CdNRs, with magnified views suggesting growth *via* linear fusion or oriented attachment of smaller CdNPs. The insets highlight contrast variations and fused domains, providing successive magnifications that support the proposed nucleation-assembly mechanism.

Building on TEM evidence of sMPC-Cd's morphological evolution from spheres to rods, a complementary xylenol orange (XO) metallochromic assay quantitatively confirmed its strong Cd^2+^ chelation capacity (Fig. 9A and B).^2^ Spectrophotometry revealed a dose-dependent absorbance decrease at 575 nm as sMPC displaced Cd^2+^ from the XO–Cd^2+^ complex, achieving >95% chelation efficiency, while a vivid color shift from violet to yellow–orange provided a rapid visual indicator of metal removal.^42,43^ These data collectively demonstrate that sMPC functions as a multidentate, biomimetic ligand capable of out-competing a classical metallochromic chelator, corroborating the structural evidence for dense, cooperative binding sites observed in TEM. This convergence of spectroscopic and microscopic findings underscores sMPC's exceptional selectivity, biocompatibility, and translational potential for real-time cadmium detoxification in environmental and biomedical contexts (Fig. 9A and B).^13^

**Fig. 9:**
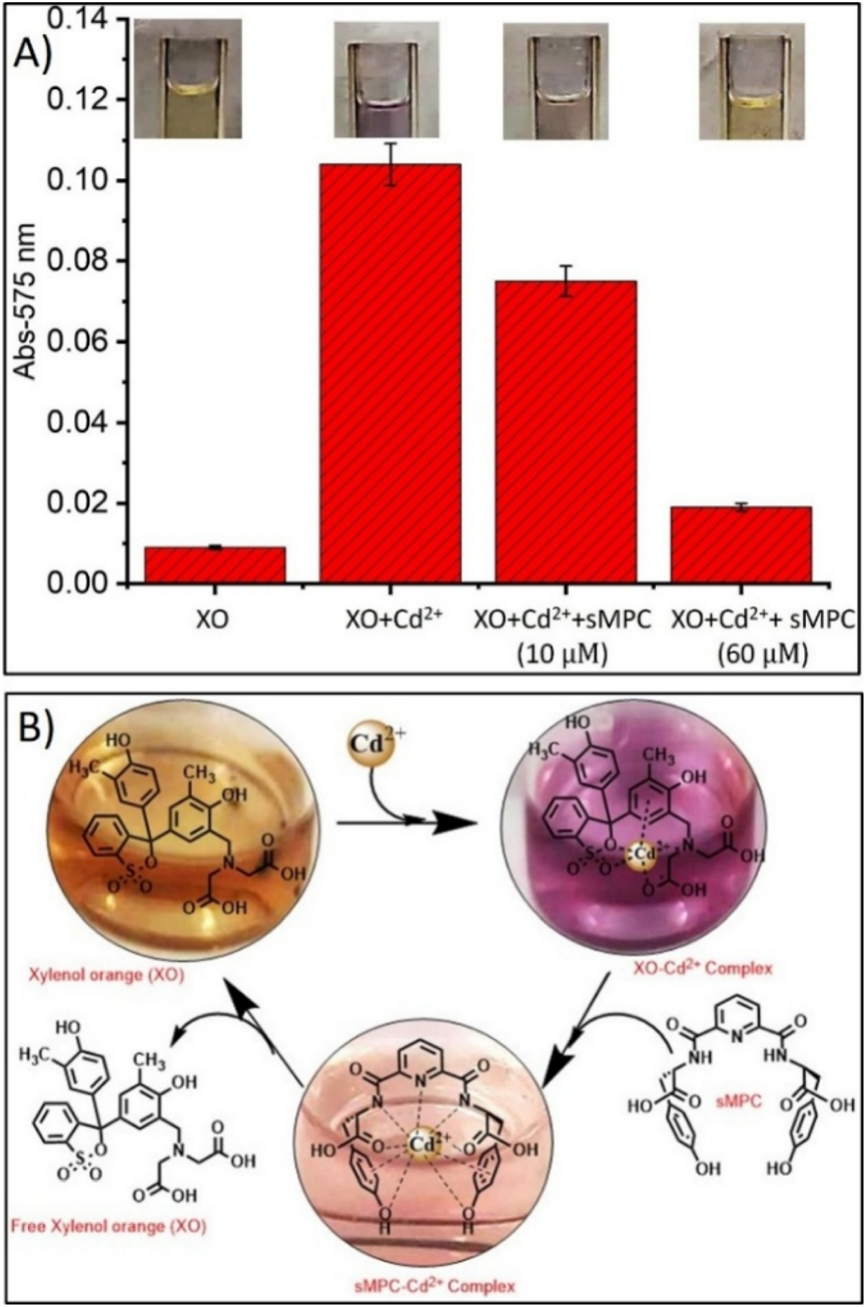
Depicts (A and B) cadmium (Cd^2+^) chelation by the short metallopeptide conjugate (sMPC) was evaluated using a xylenol orange (XO)-based metallochromic assay. XO forms a violet XO–Cd^2+^ complex with strong absorbance at 575 nm. Increasing sMPC concentrations (10–60 µM) progressively displaced Cd^2+^, reducing absorbance by >95% at higher levels (≥30 µM) and producing a visible colour shift from violet to yellow/orange. This displacement reflects multidentate coordination by sMPC *via* hydroxyl, amide and carboxylate groups, consistent with its schematic representation (B). Together these results confirm sMPC's high metal-binding affinity and highlight its promise as a biomimetic agent for cadmium detoxification.

Because HEK-293 cells are human embryonic kidney-derived and kidneys are primary targets of cadmium accumulation and toxicity, this cell line serves as a highly relevant *in vitro* model for assessing cadmium-induced cytotoxicity and evaluating the protective effects of potential chelating agents. To rigorously assess the biocompatibility and therapeutic potential of sMPC, its cytotoxicity was first evaluated in HEK-293 cells using the MTT assay (Fig. 10A).^13^ As expected, untreated controls displayed maximal viability, reflecting intact metabolic activity and membrane integrity. In contrast, cells exposed to Cd^2+^ alone showed a marked viability reduction, confirming the well-documented cytotoxicity of cadmium ions.^13^ Remarkably, co-treatment with sMPC in the presence of Cd^2+^ significantly restored cell viability, underscoring its ability to chelate cadmium and mitigate toxic effects.^2^ Treatment with sMPC alone produced negligible cytotoxicity, highlighting a favourable biocompatibility profile (Fig. 10A). These findings, in agreement with prior spectroscopic and microscopic analyses, establish sMPC as a potent, safe, and multifunctional chelating scaffold with promising biomedical applications.^13,17,24^

**Fig. 10:**
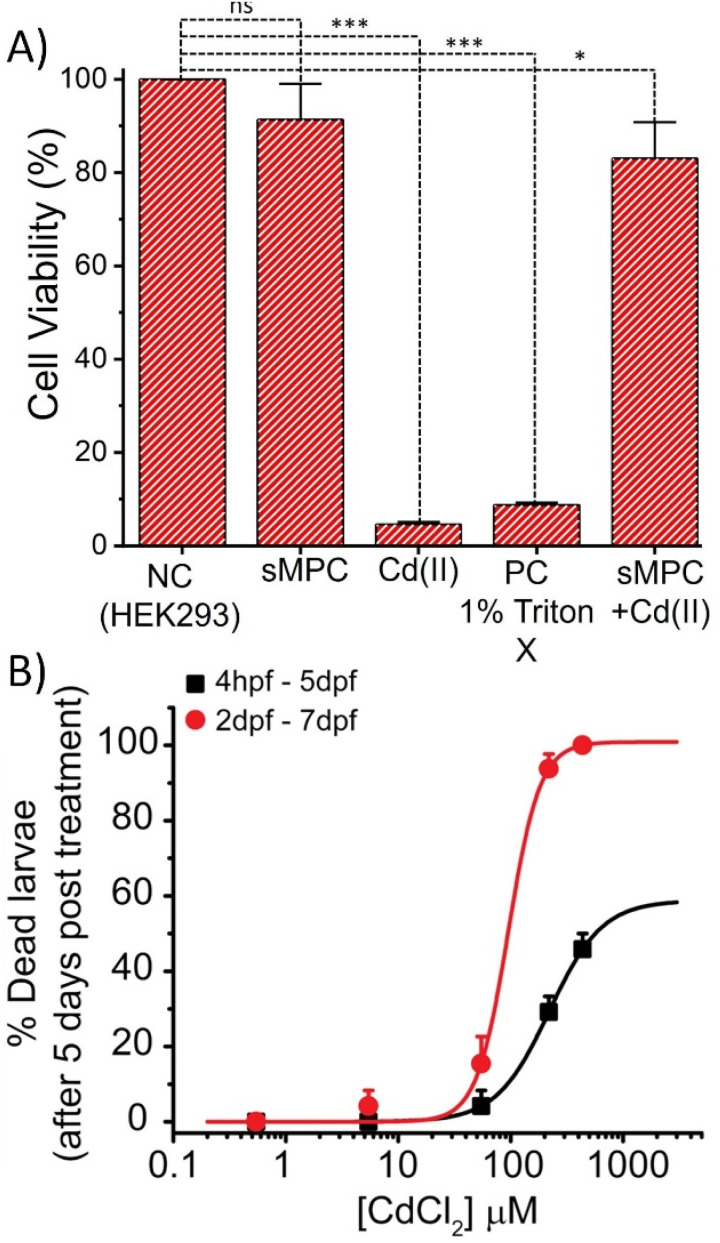
Depicts (A) MTT assay shows % cell viability for each treatment. Statistical analysis reveals significant differences among the five groups (NC, PC, sMPC, sMPC + Cd, and Cd), indicating a strong treatment effect. ***P < 0.0001, *P < 0.05. (B) Dose-dependent effect of CdCl_2_ on zebrafish larvae survival. Hill's equation-fitted curves show mortality after five days of exposure from 4 hpf (black squares, LC_50_ = 220.1 ± 1.2 µM) and from 2 dpf (red circles, LC_50_ = 95.03 ± 10.5 µM).

Cadmium (Cd^2+^), a widespread environmental contaminant, has been extensively linked to developmental and neurotoxic defects across model organisms, including zebrafish (*Danio rerio*).^4,44^ Exposure during early embryogenesis produced hallmark deformities such as delayed hatching, cranial and cardiac edema, trunk malformations, and reduced survival, outcomes consistent with earlier reports.^44–46^ At sub-micromolar concentrations, CdCl_2_ exposure induced changes in tissue transparency without overt malformations, suggesting that optical changes may represent an early biomarker of Cd-induced toxicity. These developmental abnormalities are attributable to cadmium's disruption of neurotransmission, synaptic plasticity, and oxidative balance, processes essential for neural function and organismal fitness.^44,45^

The conservation of these toxic phenotypes in mammalian systems underscores the translational significance of zebrafish as a model to assess cadmium toxicity and evaluate protective interventions.^46^ Zebrafish embryos develop within a protective chorion that ruptures around 48 hpf to release free-swimming larvae (Fig. 10B). Exposure of embryos to CdCl_2_ from ∼4 hpf to 5 dpf produced a dose-dependent lethality with an LC_50_ of 220.1 ± 1.2 µM, though 100% mortality was not reached even at >400 µM (80 mg L^−1^). This resistance is consistent with the chorion acting as a barrier that limits cadmium uptake. In contrast, larvae exposed post-hatching (2–7 dpf) showed an LC_50_ of 95.03 ± 10.5 µM, with complete mortality at >400 µM, indicating higher vulnerability once the chorion is lost. Reported cadmium LC_50_ values in zebrafish range from <20 to >200 µM, depending on strain, developmental stage, exposure conditions, and duration (Fig. 10B). Our results therefore validate the protective role of the chorion during early embryogenesis and fall within the spectrum of published toxicity data.^46^

The conservation of these toxic phenotypes in mammalian systems underscores the translational significance of zebrafish as a model to assess cadmium toxicity and evaluate protective interventions.^46^ Before evaluating the therapeutic role of the peptide against cadmium toxicity, it was essential to validate the effect of the experimental conditions themselves, particularly the use of 0.32% ethanol (as solvent control) and the peptide (sMPC, 16.3 µM). Morphological analyses of zebrafish larvae under these conditions (Fig. 11A) revealed no gross developmental abnormalities when compared with the untreated control group. Quantitative assessment of four key morphological biomarkers-eye length (EL), half-body length (HBL), abdominal length (AL), and thoracic length (TL)-further confirmed the absence of significant changes (Fig. 11B–E). In all cases, values for the ethanol- and peptide-treated groups were statistically indistinguishable from those of the control group, as indicated by “n.s.” (not significant).

**Fig. 11:**
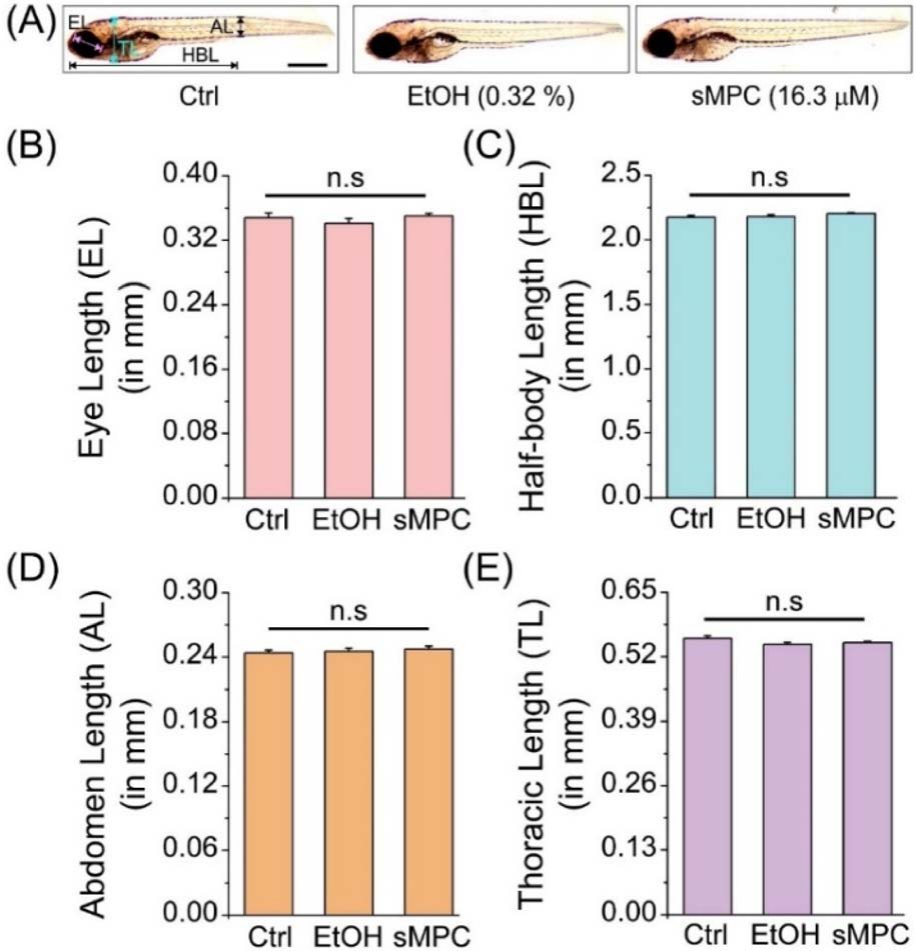
Morphometric analysis validating zebrafish larval biocompatibility under experimental conditions. (A) Representative images of control (Ctrl), ethanol (EtOH 0.32%, solvent control), and sMPC (16.3 µM) groups; control larvae show reference morphometric markers (scale bar = 0.5 mm). (B–E) Quantitative measurements of eye length (EL), half-body length (HBL), abdominal length (AL), and thoracic length (TL) from 2–5 dpf revealed no significant differences (n.s.) *versus* buffer controls. Data are mean ± SEM (18–21 larvae, two trials). Both ethanol and sMPC treatments showed no morphological abnormalities, confirming biocompatibility for cadmium rescue studies.

These results demonstrate that both the solvent condition and the working concentration of sMPC are biocompatible and non-toxic to zebrafish embryos, even during continuous exposure from early embryogenesis through larval stages. This validation step is critical, as it ensures that any morphological or developmental rescue effects observed in subsequent cadmium co-exposure experiments can be attributed specifically to the therapeutic action of sMPC, rather than confounding effects of ethanol or the peptide itself. The findings thus establish a robust experimental baseline for assessing peptide-mediated protection against cadmium-induced toxicity.

To assess the therapeutic efficacy of our synthetic metallopeptide (sMPC), zebrafish larvae were exposed to 54.5 µM CdCl_2_ from ∼4 hpf to 5 dpf, a concentration sufficient to elicit consistent developmental toxicity. Treatment with 16.3 µM sMPC was initiated post-hatching (after 2 dpf) and continued in the presence of cadmium, thereby simulating a therapeutic rather than preventive scenario. Morphometric analysis revealed significant cadmium-induced impairments in eye length, half-body length, and abdominal length, while thoracic length remained unaffected (Fig. 12B–E). Remarkably, sMPC treatment substantially alleviated these defects (Fig. 12A), with abdominal enlargement fully reversed to control levels (Fig. 12C). Control experiments with ethanol (0.32%, solvent) and peptide alone demonstrated no significant differences relative to untreated controls, confirming that the peptide is biocompatible and does not perturb normal development (Fig. 12A and 11). Collectively, these results provide the first *in vivo* evidence of sMPC's ability to mitigate cadmium-induced teratogenicity.

**Fig. 12:**
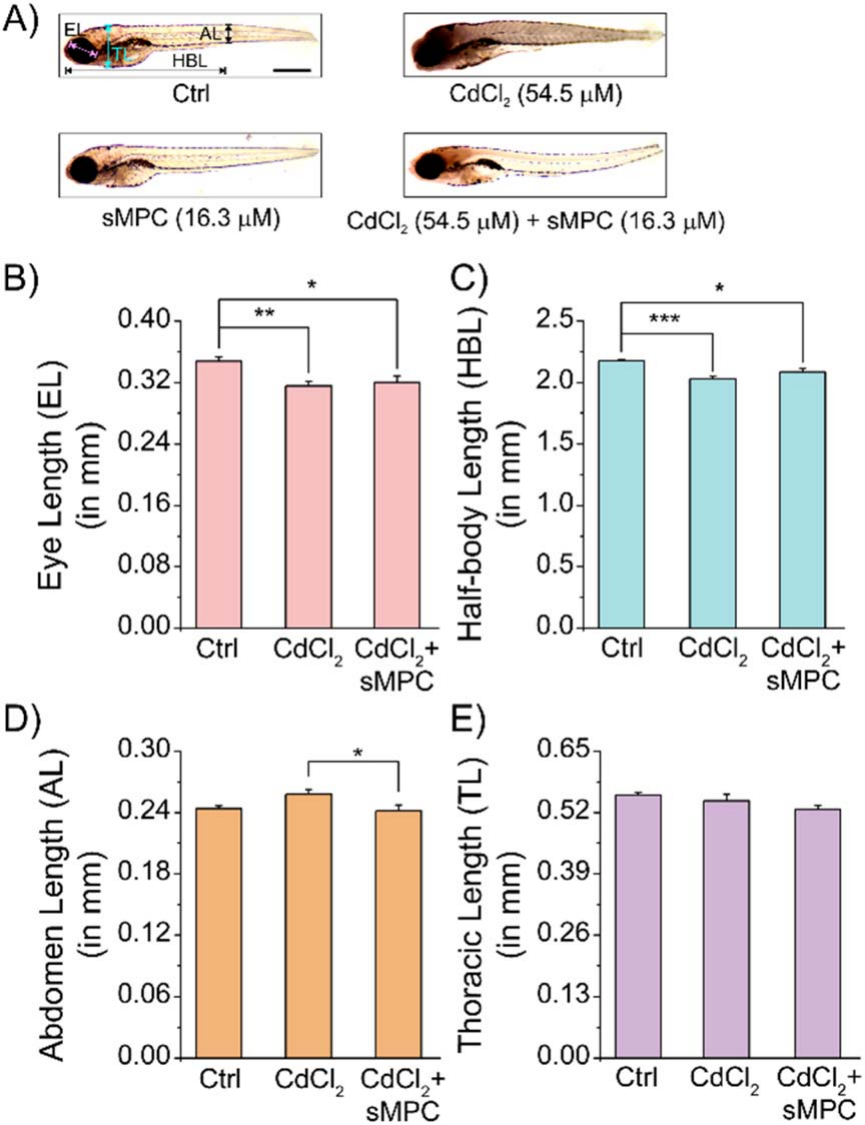
Morphometric analysis of zebrafish larvae treated with CdCl_2_ and sMPC. (A) Representative images of control (Ctrl), CdCl_2_-treated (54.5 µM), sMPC (16.3 µM) treated and co-treated (CdCl_2_ 54.5 µM + sMPC 16.3 µM post-2 dpf) groups; scale bar: 0.5 mm. Control image highlights morphometric biomarkers. (B–E) Quantification of eye, half-body, abdomen, and thoracic lengths shows CdCl_2_-induced developmental toxicity, alleviated by sMPC. Controls were in E3 buffer (∼4 hpf–5 dpf); co-treated group 1st received CdCl_2_ from 4 hpf till 5 dpf stage followed by sMPC at 2 dpf (post-hatching) till 5 dpf stage. Data: mean ± SEM (*n* = 18–21, two replicates). *P < 0.05, **P < 0.01, ***P < 0.001.

Mechanistically, cadmium toxicity is strongly associated with elevated oxidative stress markers such as malondialdehyde (MDA) and reactive oxygen species (ROS), leading to inflammation and long-term tissue dysfunction in the brain, liver, and gills.^47,48^ Our findings are consistent with these established pathways and highlight the importance of sMPC as both a chelating agent and a stabilizing scaffold. By attenuating oxidative and structural damage, sMPC provides a low-micromolar, non-toxic therapeutic strategy for cadmium detoxification *in vivo*. More broadly, the convergence of cellular assays, zebrafish developmental studies, and biochemical evidence positions sMPC as a multifunctional metallopeptide platform for environmental and biomedical applications.^9,49^ This work thus establishes proof-of-concept that rationally designed short peptides can be leveraged to combat heavy-metal toxicity, bridging the gap between *in vitro* chelation and translational therapeutic development.

## Conclusion

In conclusion, our work establishes cadmium (Cd^2+^) as both an ecological and biological threat and introduces a first-in-class, biomimetic, stimuli-responsive supramolecular metallopeptide conjugate (sMPC) as a dual-function platform for its selective detection and detoxification. These short metallopeptides display precise, high-affinity chelation of Cd^2+^ and adaptive self-assembly that converts toxic cadmium into structurally inert, Cd nanorod-like architectures (CdNRs). Combined spectroscopic, microscopic, electrochemical and computational analyses delineate the underlying metal-binding and morphological transformation mechanisms, while *in vitro* (HEK-293) and *in vivo* (zebrafish) assays demonstrate excellent biocompatibility, efficient cadmium clearance, oxidative stress reduction and tissue recovery. According to an extensive literature survey (Tables S1 and S2), this is the first peptide-based system capable of simultaneously sequestering toxic Cd^2+^ and reorganising it into nanostructured forms, thereby offering a pioneering, real-time approach for cadmium sensing and therapeutic detoxification with broad environmental and biomedical relevance.

## Author contributions

AK, SS and KBJ conceived and designed the study. SS, with input from AK, carried out the synthesis under KBJ's guidance. AK and SS performed most experiments and data analysis with supervision from KBJ. RK and BD contributed fluorescence and MTT assays, respectively, under SM's supervision. YB, AK and SS designed the zebrafish study with KBJ; AW performed experiments and YB analysed and interpreted data. NK conducted DFT calculations under PG's supervision, with interpretation discussed with KBJ. KBJ supervised all experimental and theoretical aspects and guided data interpretation. AK drafted the manuscript with input from KBJ; all authors reviewed, edited and approved the final version.

## Conflicts of interest

There are no conflicts to declare.

## Supplementary Material

NA-OLF-D5NA00969C-s001

## Data Availability

All data supporting the findings of this study are included within the article and its supplementary information (SI). Supplementary information is available. See DOI: https://doi.org/10.1039/d5na00969c.
