# Supplementary material for: Short metallopeptide conjugate nanostructures for selective cadmium capture and detoxification
Source: Nanoscale Adv. 2025 Dec 3;8(1):309–18. doi: 10.1039/d5na00969c (PMC12687284; doi:10.1039/d5na00969c)
Supplement: NA-008-D5NA00969C-s001 [file NA-008-D5NA00969C-s001.pdf]

Supplementary Information (SI) for Nanoscale Advances.
This journal is © The Royal Society of Chemistry 2025

Electronic Supplementary Information

1. Materials and Methods (General): All chemicals and solvents used in this study were of analytical or HPLC grade and procured from reputed commercial suppliers, in accordance with the specific requirements of each experiment. Unless stated otherwise, all reagents were used as received, without further purification. L-Tyrosine and thionyl chloride were purchased from Spectrochem Pvt. Ltd. (Mumbai, India). The linker molecule 2,6-pyridinedicarboxylic acid and the dye Xylenol Orange were obtained from SRL Pvt. Ltd. (India). Peptide coupling agents, including N-hydroxybenzotriazole (HOBt) and 1-ethyl-3-(3-dimethylaminopropyl) carbodiimide hydrochloride (EDC·HCl), were sourced from Avra Synthesis Pvt. Ltd. (Hyderabad, India). Organic bases such as triethylamine (Et_3N), diisopropylethylamine (DIPEA), and 4-dimethylaminopyridine (DMAP) were supplied by S D Fine-Chem Ltd. (India). Silica gel (mesh sizes 60–120 and 100–200) and TLC plates (Silica gel 60 F254 on aluminum backing) were procured from Merck Chemicals (India). Common solvents such as methanol, ethanol, dichloromethane, ethyl acetate, acetone, chloroform, dimethyl sulfoxide (DMSO), diethyl ether, acetonitrile, pyridine, N,N-dimethylformamide (DMF), and tetrahydrofuran (THF) were also obtained from S D Fine-Chem Ltd. HPLC-grade solvents and deuterated NMR solvents were sourced from Merck Pvt. Ltd. (India). Metal salts were purchased from HiMedia Laboratories Pvt. Ltd. (Mumbai, India). All other reagents and materials used in this study were also obtained from HiMedia, unless otherwise specified.

2. Peptide Synthesis- The synthesis of the C_2-Symmetric Pyridine-bis-Tyrosine short metallopeptide conjugate sMPC was performed by conventional solution phase synthesis via established lab protocol mentioned in research paper published our group. [1–3] The purity and identity of these compounds were confirmed before their utilization. All experiments were conducted under ambient room temperature conditions.

3. UV-Vis Spectroscopic Studies: UV-Vis absorption spectra were recorded using a Lab India UV-VIS Spectrophotometer 3000+, equipped with a 10 mm quartz cuvette, and maintained at a

stable temperature of 25 ± 0.1°C. Initially, a 1 mM stock solution of each metal ion was carefully prepared in deionized water. Subsequently, solutions containing 10 µM concentrations of short Metallopeptide Conjugate (sMPC) in ethanol were titrated with the corresponding metal ions, incrementally increasing the concentration to 70 µM, utilizing the prepared stock solutions.[1–4]

4. Fluorescence Spectroscopy- Fluorescence intensity was measured at room temperature using an excitation wavelength of 260 nm. The interactions between sMPC and the relevant metal ions were studied individually to observe any changes in their behavior. The emission spectra were collected from 280 nm to 500 nm. For this, a stock solution of each metal ion salt was prepared at a concentration of 15 mM in water. The peptide solution (20 µM) was then gradually added to the metal ion solutions, reaching a final concentration of 45 µM. Fluorescence measurements were performed using a Varian Luminescence Cary Eclipse spectrophotometer with a 10 nm quartz cell, keeping the temperature steady at 25 ± 0.1°C. All solutions were made using high-purity water and ethanol.

5. FT-IR Study- Fourier-transform infrared (FT-IR) spectroscopy was utilized to examine the functional group composition of both the sMPC peptide and its complex with metal ions. The spectra were recorded using a Bruker Alpha II attenuated total reflection (ATR) instrument. Spectral data were collected in the range of 4000 to 500 cm^{-1}, with a resolution of 4 cm^{-1}, employing 2 sample gain and 32 sample/background scans to ensure accurate measurements. The data were processed using OPUS 7.0 software, which included noise reduction procedures for clearer spectral output.

For the study, a 250 µM solution of sMPC was prepared, both in the absence of metal ions and in the presence of 1 mM Cd^{2+} ions, at a 1:3 metal-to-peptide ratio. These solutions were incubated for 12 and 24 hours, respectively. After incubation, the solutions were applied to a ZnSe substrate and allowed to dry. The Amide I region (1700–1600 cm^{-1}) was then analysed using deconvolution via Origin software. The deconvolution process was guided by the initial peak values derived from the second derivative spectrum, helping to resolve overlapping bands.

Quantitative analysis of the areas associated with each spectral component was performed to evaluate how metal ion interaction influences the secondary structure of the sMPC peptide[5].

6. NMR Spectroscopy and NMR titration Experiments: Samples were prepared by dissolving the compound in deuterated solvents (*DMSO-d_6*). ^{1}H and ^{13}C-NMR spectra were recorded at 25°C on JEOL-JNM ECS 400 and 500 MHz JEOL ECX spectrometer. 1D spectrum was recorded at a peptide concentration of 10 mg/400µL in DMSO-d_6 at 298 K.[5-6]

7. Transmission Electron Microscopy (TEM): In accordance with established protocols for Transmission Electron Microscopy (TEM) sample preparation, a freshly prepared 10 µL solution of 200 µM concentration was meticulously dispensed onto a copper grid that had been coated with a carbon layer featuring a mesh size of 200. Any surplus sample was delicately eliminated from the grid, and the residual specimen was permitted to undergo an air-drying process at room temperature for 6 hours. The samples were subsequently subjected to examination utilizing a FEI Titan G2 60-300 Transmission Electron Microscope (TEM). [4-6]

8. Computational Studies/ Details:

All calculations were performed using the ORCA 5.03 quantum chemical program package. Geometries were optimized with the hybrid density functional B3LYP in conjunction with def2-SVPbasis sets. To accelerate the overall calculations, the RIJCOSX approximation was applied for the expensive integral calculations. Non-covalent interactions were accounted for by using atom-pair-wise dispersion corrections with Becke-Johnson (D3BJ) damping. Subsequent numerical frequency calculations were undertaken for the optimized geometries to confirm that they correspond to stationary points featuring no imaginary frequencies. Binding energies are calculated by single-point calculations with B3LYP/def2-TZVPCPCMEtOH) method on the B3LYP/def2-SVP geometries.[7-13]

The binding energy of the metal-peptide complexes was calculated using the equation:

$$Binding\ Energy = G_{MPy(Tyr)_2} - (G_M + G_{Py(Tyr)_2})$$

Where $\mathbf{G_{MPy(Tyr)2}}$, $\mathbf{G_M}$ and $\mathbf{G_{MPy(Tyr)2}}$ are the Gibbs energy of the metal-peptide complex, metal and the peptide fragment, respectively.

9. Vitro Cytotoxicity Assessment of Cd(II) Detoxification Mediated by sMPC Using Cell Viability Assay: To assess the cytotoxic profile and detoxification efficacy of the synthesized short metallopeptide conjugate (sMPC), an in vitro MTT assay was conducted on human embryonic kidney (HEK293) cells. The assay compared the effects of sMPC alone, cadmium (II) ions and the sMPC–Cd(II) complex. HEK293 cells were seeded at a density of 0.2×10^6 cells per well in a 12-well tissue culture plate and incubated overnight at 37 °C in a 5% CO_2 atmosphere to allow for proper adherence and recovery. Post incubation, cells were treated with 100 nM concentrations of sMPC, Cd(II), or their conjugate. Untreated cells were included as a negative control to establish baseline viability. Cytotoxicity was assessed using the EZ Count MTT Cell Assay Kit (Himedia, Cat. No. CCK003), following the manufacturer's protocol. After incubation with MTT reagent, absorbance was recorded at 540 nm using a BioTek Epoch Microplate Spectrophotometer. Cells treated with 2% Triton X-100 served as a positive control, demonstrating complete loss of viability, whereas the untreated control group showed maximum viability.[4,14]

10. Zebrafish Culture and Embryo Production: Adult zebrafish (both males and females) were obtained from a local vendor and subjected to a quarantine period of 2-3 weeks in isolated tanks. Following quarantine, the zebrafish were transferred to a stand-alone Open-RAC setup, as previously described.[16-18] The fish were maintained at 30 °C with a 14/10-hour light/dark photoperiod and fed ad libitum twice daily with commercial fish pellets. Fertilized embryos were obtained using standard operating procedures and stored in 1X E3 medium containing (in M): 0.0595 NaCl, 0.021 KCl, 0.039 $CaCl_2 \cdot 2H_2O$, and 0.048 $MgCl_2 \cdot 6H_2O$, adjusted to pH 7.2, as described previously. All animal handling and experiments were conducted in accordance with the guidelines prescribed by the Committee for the Purpose of Control and Supervision of Experiments on Animals (CPCSEA), Government of India.

11. Treatment Solutions: A stock solution of 5 mg/mL $CdCl_2$ was prepared in water. Serial dilutions of this stock were then made in 1X E3 media to achieve final $CdCl_2$ concentrations of 0.54 µM, 5.45 µM, 54.5 µM, 218.34 µM, and 436.68 µM in a total volume of 10 mL. Separately, a 5 mM stock solution of the sMPC was prepared in ethanol. Immediately prior to the experiment, the sMPC stock was diluted in 1X E3 media to obtain a final concentration of 16.3 µM in a total volume of 10 mL. We used 0.32 % ethanol in 1X E3 media as a solvent control and 1X E3 media alone (without $CdCl_2$, sMPC, or ethanol) as a ligand control.[19]

12. Determination of half-maximal lethal concentration of $CdCl_2$ for zebrafish larvae: The half-maximal lethal concentration (LC_{50}) of $CdCl_2$ for zebrafish larvae was determined following a modified protocol based on previous methods[20]. Briefly, in each experimental trial, ten to twelve zebrafish embryos at ~4 hours post-fertilization (hpf) stage were exposed to various concentrations of $CdCl_2$ in a static mode until 5 days post-fertilization (dpf) stage. Dead embryos were manually removed from both control and $CdCl_2$-treated groups every 24 hours under a dissecting microscope. Heartbeat was used as the criterion to distinguish live from dead embryos. Data were plotted for each treatment group as the mean percentage ± SEM of cumulative dead embryos, across 2–4 biological trials over the exposure period. To assess the protective role of the chorion layer in unhatched zebrafish larvae against $CdCl_2$-induced toxicity, LC_{50} was also determined using a similar protocol. In this variation, $CdCl_2$ exposure began after 2 dpf stage and continued until 7 dpf stage, maintaining a total exposure duration of 5 days. This approach allowed for comparison between embryos exposed to $CdCl_2$ starting at ~4 hpf stage and those exposed after the chorion was naturally shed during hatching i.e., after 2 dpf stage.

13. Determination of $CdCl_2$ toxicity and therapeutic effect of sMPC on developing zebrafish larvae: For each treatment group, ten to twelve zebrafish larvae at ~4 hours post-fertilization (hpf) were continuously exposed to 54.5 µM $CdCl_2$ until 5 days post-fertilization (dpf). Ethanol (0.32 %) or sMPC (16.3 µM) treatments were initiated after 2 dpf stage and continued until 5 dpf stage. All exposures were conducted in a static mode, with no solution exchanges throughout the

experiment. Developing zebrafish were maintained at 28 °C for the entire duration of treatment. Data are presented as the mean ± SEM for each treatment group, based on two independent biological trials.

14. Statistical Analysis: Data represented in each figure related to zebrafish biomarkers for evaluating birth defects are expressed as the mean ± SEM, based on 18–21 zebrafish larvae from two independent biological trials. Statistical significance between treatment groups was assessed using one-way ANOVA followed by Tukey's post hoc test, performed with GraphPad Prism v5.01. A P-value of < 0.05 was considered statistically significant, with significance levels indicated in the figures as follows: *$P < 0.05$, **$P < 0.01$, ***$P < 0.001$.

15. Figures:

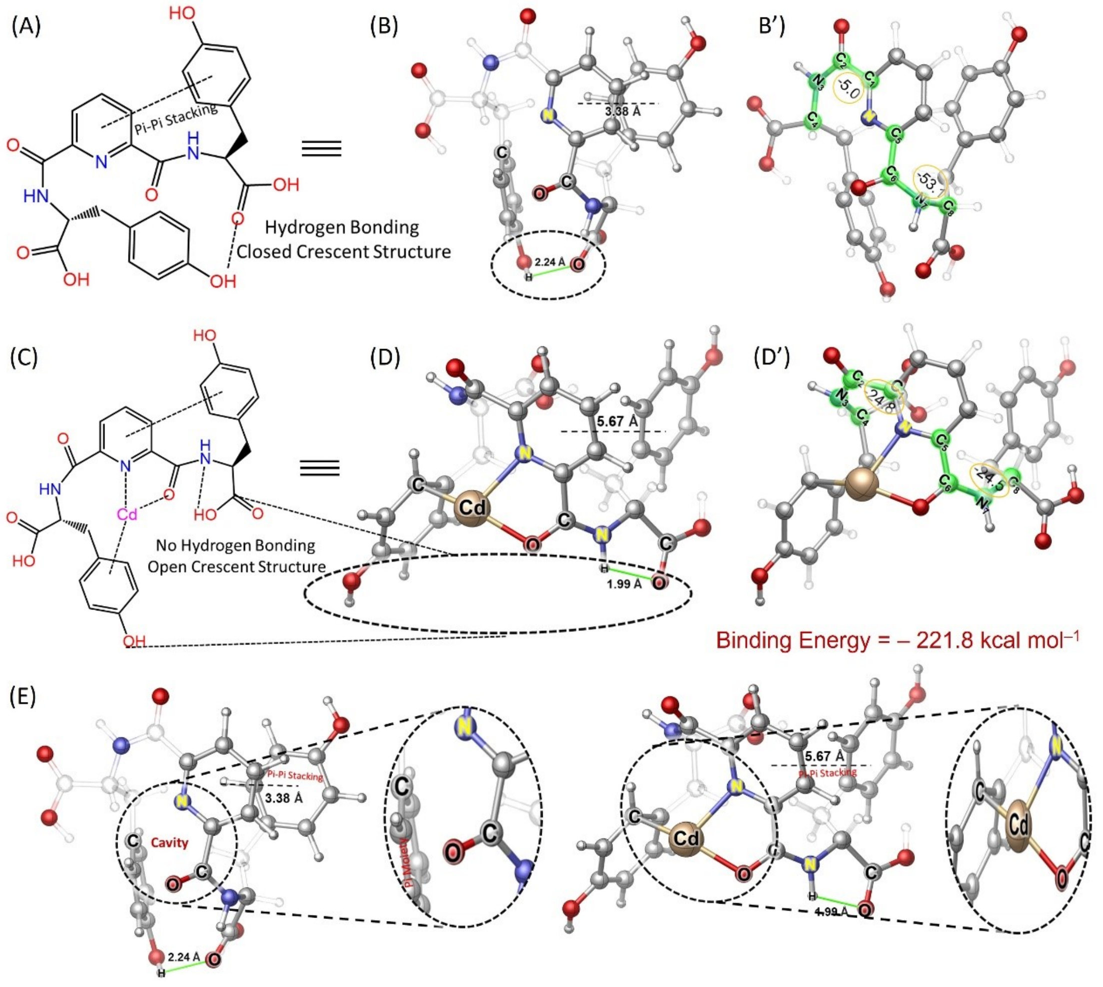

Figure S1: The native sMPC adopts a hydrogen-bond-stabilized, closed crescent-like conformation (A), facilitated by intramolecular hydrogen bonding (2.24 Å) and aromatic π–π stacking (3.38 Å). This structure is validated by DFT-optimized geometry (B), which preserves the key non-covalent interactions, and further supported by torsional angle analysis in (B′), confirming preorganized rigidity. Upon Cd(II) binding, hydrogen bonding is disrupted, inducing a pronounced conformational shift to an open crescent structure (C), as revealed by DFT-optimized Cd(II)-bound structure (D). The metal ion coordinates with pyridinic nitrogen and backbone carbonyl oxygen atoms, forming strong coordination bonds and increasing the inter-ring distance to 5.67 Å. Torsion angle variation (D′) and reflects this transition, highlighting structural adaptability.Panel (E) presents an overlay of unbound and Cd(II)-bound geometries, illustrating

the formation of a selective, well-defined binding cavity (highlighted in insets) that spatially orients donor atoms for chelation. The highly favorable binding energy (−221.8 kcal mol⁻¹) underscores strong thermodynamic stability, demonstrating the molecular design's promise for cadmium detoxification via selective metal sequestration.

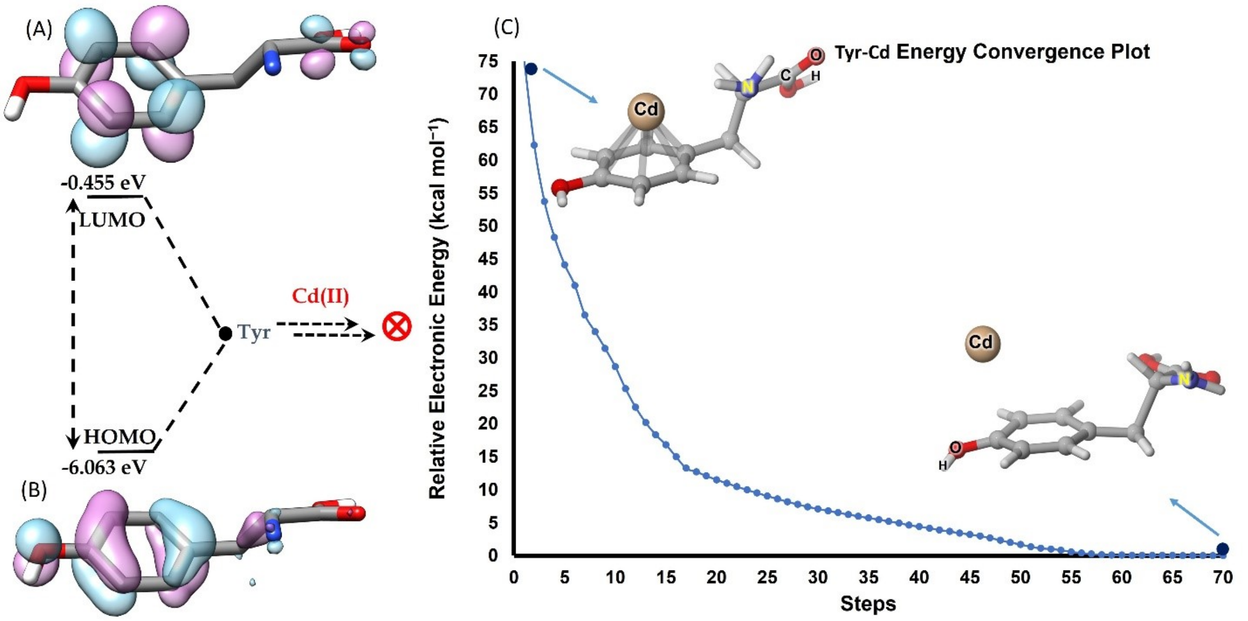

Figure S2: Frontier molecular orbital (FMO) analysis and energy convergence profile revealing the non-coordination behavior of Cd^{2+} toward tyrosine.(A, B) DFT-calculated HOMO and LUMO surfaces of tyrosine (B3LYP/def2-SVP level) indicate well-localized electronic distributions in the absence of Cd^{2+}. Upon attempted Cd^{2+} introduction, no significant interaction between tyrosine and Cd is observed, implying the absence of favorable electronic interaction or binding affinity between tyrosine and Cd^{2+}. (C) The relative electronic energy convergence plot further supports this observation, showing a progressive increase in system stability as the Cd^{2+} ion is displaced from the tyrosine environment. The lack of convergence to a stable coordination geometry, coupled with repulsion-driven energy minimization, confirms that tyrosine lacks the spatial and electronic compatibility to chelate Cd^{2+}, resulting in its exclusion from the molecular environment. This behavior highlights the importance of multivalent peptide frameworks (such as sMPC) in achieving selective metal coordination, which isolated tyrosine residues fail to accomplish.

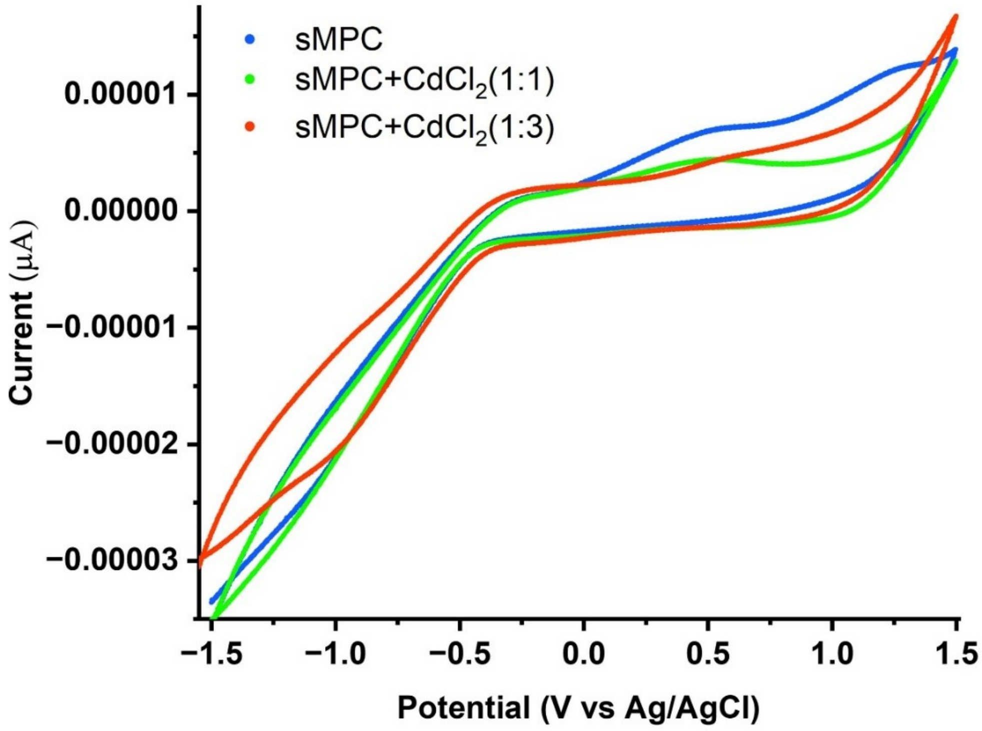

Figure S3: Cyclic voltammetry (CV) analysis revealing the electrochemical response of sMPC and its interaction with Cd^{2+} ions.CV profiles of sMPC (blue), sMPC + $CdCl_2$ (1:1, green), and sMPC + $CdCl_2$ (1:3, red) were recorded to investigate metal-induced changes in redox activity. The pristine sMPC displayed two prominent oxidation peaks around +0.5 V and +1.2 V (vs Ag/AgCl), attributed to the oxidation of phenolic –OH groups of tyrosine residues. Upon incremental addition of Cd^{2+}, these peaks were progressively suppressed, with the peak at +1.2 V diminishing more rapidly, indicating perturbation of the tyrosine redox environment due to coordination with Cd^{2+}. At higher Cd^{2+} equivalents (1:3), both oxidation peaks vanished, suggesting the formation of a stable sMPC-Cd^{2+} complex. This likely results from reduced electron transfer kinetics owing to molecular aggregation or steric hindrance, supporting the role of sMPC in effective cadmium sequestration via multivalent binding.

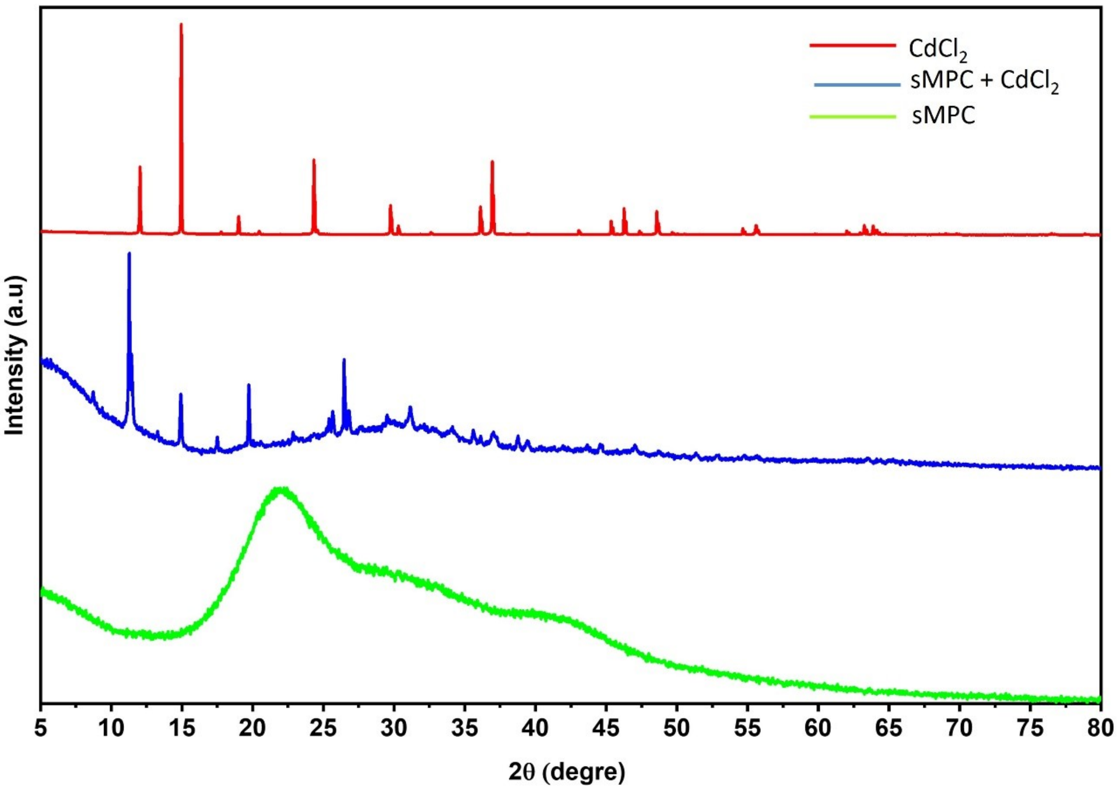

Figure S4: Powder X-ray diffraction (XRD) patterns of $CdCl_2$ (red), self-assembled short metallopeptide conjugate (sMPC, blue), and sMPC incubated with $CdCl_2$ (green). $CdCl_2$ displays sharp and well-defined Bragg reflections indicative of its crystalline nature. In contrast, the sMPC exhibits a amorphous peptide-based assemblies, consistent with TEM and other reports of short peptide-derived nanostructures deposited on glass substrates. Upon coordination with Cd^{2+} ions, the resulting sMPC–Cd complex shows significant attenuation of $CdCl_2$ crystalline peaks, alongside a broader, less-defined diffraction profile. This transition suggests disruption of long-range order and incorporation of Cd^{2+}into the sMPC matrix, potentially forming an amorphous coordination network. The data supports effective chelation and morphological transformation of sMPC nanostructures in the presence of Cd^{2+}.

16. Determination of Cadmium Chelation Efficiency of sMPC using Xylenol Orange (XO) Metallochromic Assay: The cadmium-chelating ability of the short metallopeptide conjugate (sMPC) was assessed using a colorimetric displacement assay based on the metallochromic indicator xylenol orange (XO). XO forms a purple complex with Cd^{2+}, characterized by a distinct absorbance at 575 nm. This spectral signature arises from charge-transfer interactions between Cd^{2+} and the sulfonate/carboxylate moieties of XO (Fig S7 &S8).Upon titration of the XO–Cd^{2+} complex with increasing concentrations of sMPC (from 10&60 µM), a gradual decrease in absorbance at 575 nm was observed, along with a visible shift in solution color from violet to pale yellow. This reflects the competitive displacement of $Cd^{2+}$from the XO–Cd^{2+} complex by the sMPC ligand, which forms a more thermodynamically favorable sMPC– Cd^{2+} complex via coordination through phenolic, carboxylate, and amide functionalities.

The chelation efficiency was calculated using the following formula:

$$Chelation\ Efficiency\ (\%) = \left(\frac{\{A_0 - A_x\}}{\{A_0 - A_b\}}\right) \times 100$$

Where:

- A_0 = Absorbance of XO–Cd^{2+}complex (control)
- A_x = Absorbance after addition of sMPC ($A_{sMPC\text{-}XO\text{-}\ Cd(II)}$)
- A_b = Baseline absorbance of free XO

As illustrated in Figure S7, the chelation efficiency increased dose-dependently with sMPC, reaching a maximum displacement at the 1:3 ratio. This demonstrates the strong binding affinity of sMPC towards Cd^{2+}, confirming its potential as a biomimetic chelator for heavy metal detoxification applications. The accompanying inset photographs further validate the chromogenic response, showing a distinct colour fade correlating with reduced absorbance values.[21,22]

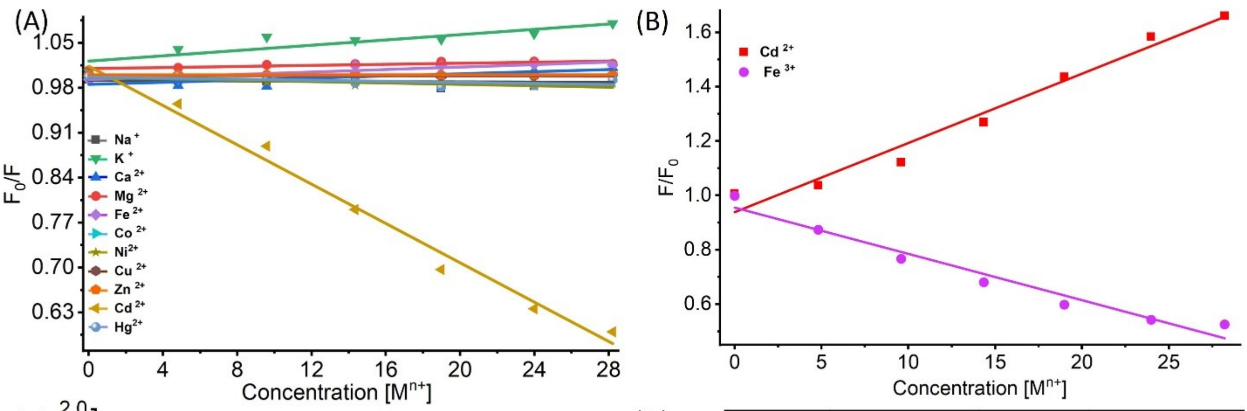

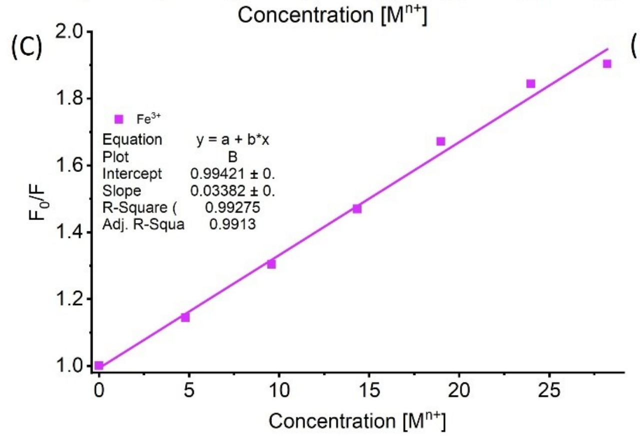

(D)

Metals	K_{sv} (LMol^{-1})	Metals	K_{sv}(LMol^{-1})
Na^+	0.120×10^3	Co^{2+}	0.0119×10^3
K^+	0.002×10^3	Ni^{2+}	0.543×10^3
Ca^{2+}	0.827×10^3	Cu^{2+}	0.0453×10^3
Mg^{2+}	0.420×10^3	Zn^{2+}	0.0065×10^3
Fe^{2+}	0.968×10^3	Cd^+	15.320×10^3
Fe^{3+}	17.04×10^3	Hg^{2+}	4.20×10^3

Comparative analysis of binding constant with different metals

Figure S5: (A) Stern–Volmer plots of sMPC with various metal ions show minimal fluorescence change for most ions, whereas Cd^{2+} induces a distinct negative slope, characteristic of chelation-enhanced fluorescence (CHEF) due to formation of a rigid Cd–ligand complex that suppresses nonradiative decay. (B) F/F_0 titrations highlight the detection response of opposite photophysical signatures of Cd^{2+} (fluorescence enhancement) and Fe^{3+} (paramagnetic quenching), enabling clear discrimination between coordination-induced rigidification and electron-transfer-mediated deactivation. (C) Linear Stern–Volmer fitting for Fe^{3+} (R^2 = 0.99275) confirms a classical dynamic quenching process. (D) Comparison of Stern–Volmer constants (K_{sv}) underscores the exceptional affinity of sMPC for Cd^{2+}, whose K_{sv} (15.320 × 10^3 L·mol^{-1}) far exceeds all other ions, establishing sMPC as a highly selective and sensitive Cd^{2+} chelating probe.

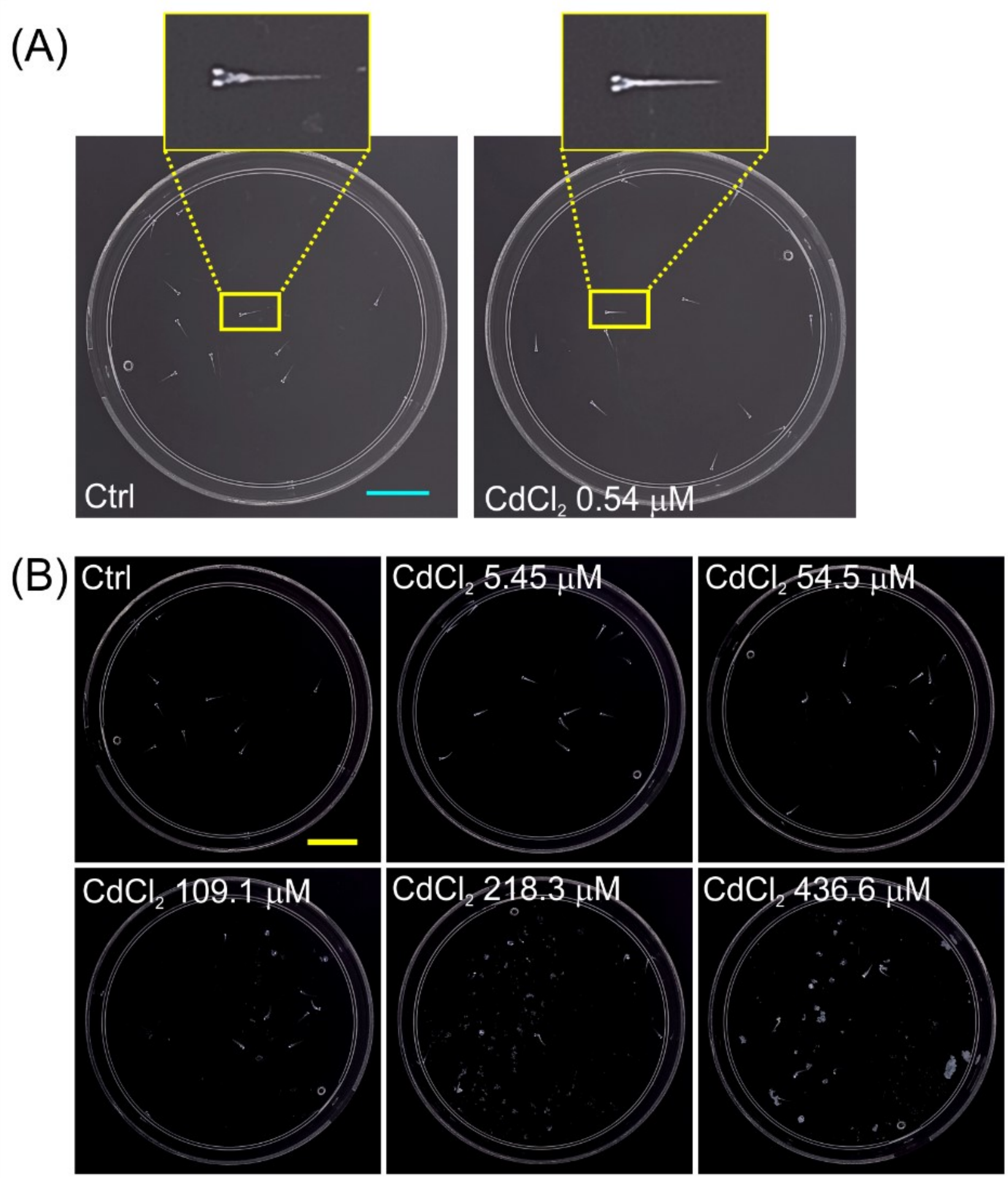

Figure S6: Depicting dose-dependent toxic effects of $CdCl_2$ on zebrafish larvae. (A) Comparative images of zebrafish larvae in control and 0.54 µM $CdCl_2$-treated groups (0.1 mg/L). (B) Comparative images of control and $CdCl_2$-treated groups exposed to 5.45 µM (1 mg/L), 54.5 µM (10 mg/L), 109.1 µM (20 mg/L), 218.3 µM (40 mg/L), and 436.6 µM (80 mg/L) concentrations of $CdCl_2$. In all images, pixel values are inverted to enhance visualization of the effects of $CdCl_2$ on the soft tissues of developing zebrafish larvae, including the chorion, gastrointestinal region, and muscles. A single larva from the control group and the lowest $CdCl_2$ concentration group (as

shown in panel A) is provided in a zoomed-in view for greater clarity. In the control group, the relative transparency or pale coloration of soft tissues indicates normal physiological development. Conversely, the increased, dense white coloration of these tissues in $CdCl_2$-treated larvae signifies tissue damage. Notably, changes in chorion transparency, especially evident in zebrafish larvae exposed to higher $CdCl_2$ concentrations (as shown in panel B), further indicate significant damage to the soft tissues.

Table-S1: Offers a detailed review about cadmium based nanoparticles with their shape, size and applications.

SN.	Cd-Based NP	Functionalization	Shape	Size	Applications	Ref. No.
1	CdS NPs	Walnut shell extract	Spherical	5–35 nm	Photocatalysis, Sensors	[23]
2	CdS NPs	Tea leaf extract	Spherical	2–5 nm	Antibacterial, Photovoltaic applications	[24]
3	CdS NPs	Unspecified (Hydrothermal)	Spherical	15–25 nm	Photocatalytic degradation of pollutants	[25]
4	CdSe NPs	Halogenated alkanes	Nanosheets (sexangular/triangular)	Few nm thickness	Photovoltaic, LED applications	[26]
5	CdSe NPs	Unspecified (Hydrothermal)	Spherical	10–30 nm	Optoelectronic devices	[27]
6	Cd NPs	*Artemisia persica* extract	Hexagonal	11.2–18.6 nm	Drug delivery, Antibacterial applications	[28]
7	CdS NPs	Sol-gel/combustion methods	Pyramid-like	Height ~400 nm	Environmental monitoring, Sensors	[29]
8	CdS NPs	Sol-gel/combustion methods	Sponge-like	200–300 nm	Supercapacitors, Catalysis	[29]
9	CdS NPs	Sol-gel/combustion methods	Hexagonal discs	50–70 nm	Solar cells, Catalysis	[29]
10	CdS NPs	Sol-gel/combustion methods	Flower-like	250–300 nm	Photocatalytic degradation	[29]
11	CdS NPs	Sol-gel/combustion methods	Rosette-like	300–400 nm	Drug delivery, Sensing	[29]
12	CdS NPs	Green/hydrothermal/sol-gel/laser ablation	Spherical	6, 9, 24, 28–32 nm	Sensors, Environmental monitoring	[30]
13	CdS NPs	Hydrothermal	Stick-like	~40 nm	Photocatalysis, Environmental applications	[30]
14	CdS NPs	Sol-gel	Hexagonal platelets	19.3–22.9 nm	Photocatalysis, Sensors	[30]
15	CdS Nanorods	Citric acid (ZnS shell)	Rod-like	Length: ~50–100 nm	Photovoltaic, LED applications	[31]
16	CdS	Biomolecule-	Rod-like	Length: ~100–	Drug delivery, Photocatalysis	[32]

	Nanorods	mediated (e.g., glycine, gelatin)		200 nm		
17	CdS Nanorods	Thermolysis in HDA	Rod-like	Length: 20–100 nm	Optoelectronics, Catalysis	[33]
18	CdSe Nanorods	Ethylenediamine decomposition	Porous rod-like	Length: ~50–150 nm	Photovoltaic, Photocatalysis	[34]
19	CdSeTe/Cd ZnS/ZnS Nanorods	Te doping, CdZnS/ZnS shells	Rod-like	Length: ~30–50 nm	Photovoltaic, Bioimaging	[35]
20	CdS-ZnO Composite Nanorods	CdS coating on ZnO rods	Rod-like	Diameter: ~50 nm	Photocatalysis, Environmental monitoring	[36]
21.	sMPC-CdNPs/Cd NRs	Functionalised with Biocompatible short Metallopeptide Conjugate (sMPC)	Dot-like and Rod-like	CdNPs-5 nm CdNRs~ 2-5 µm	In-Vivo and In-Vitro Detoxification and Detection of Cd(II)	Current Work

Table-S2: Offers a detailed review about the probe (Molecules) that's used for Cd detection and its practical applications.

SN.	Probe	LOD	Methods/Techniques	Pros/Cons	Ref. No.
1.	5-(4-Aminophenyl)-10,15,20-triphenylporphyrin	73 nM	Ratiometric variations in absorption and fluorescent emission spectra	Colorimetric/fluorescent sensor for metal ions<br>Environmental monitoring<br>Onsite visual detection	[37]
2.	Conjugated Polydiacetylenes	185 nm	Based on colorimetric and fluorometric change	Naked-Eye Detection from Aqueous media	[37]
3.	(*E*)-4-hydroxy-3-(3-(4-methoxyphenyl)acryloyl)-2*H*-chromen-2-one	58.4 nM	Based on the c turn-on chelation-enhanced fluorescence (CHEF) emission and colorimetric response	Detection from Mixed aqueous–organic	[38]
4.	Bis((indol-3-yl)methylene)oxalohydrazonamide	110 nM	Based on the c turn-on chelation-enhanced fluorescence (CHEF) emission and colorimetric response	Real World Analysis	[39]
5.	ZnS quantum dots	37.8 nM	Based on turn on fluorescence of quantum dot method probes	Real time Aqueous media	[40]
6.	$CuInS_2$ quantum dots	0.19 µM	Quenching and enhancing the fluorescence of the near-infrared $CuInS_2$ QDs	Real World Analysis	[41]

7.	TAA–AgInZnS quantum dots	1.56 µM	Based on aggregation-induced emission enhancement (AIEE) of Zn-Ag-In-S quantum dots (ZAIS QDs)	Not Available	[42]
8.	InP quantum dots	0.10 µM	Detection is based on an electron transfer process between the Qdot and the ligand, and subsequent blocking of the electron transfer pathways upon exposure to Cd2+	Not Available	[43]
**Some Peptide-Based Sensor of Cd^{2+}**					
**SN**	**Probe**	**LOD**	**Methods/Techniques**	**Real world Practical Applications**	**Ref. No.**
9.	Pyrene-Cys-Gly-Pro-Cys-OH (Probe 1)	22 nM	Ratiometric Fluorescence via Self-Assembly	Self-assembling probe; detects Cd^{2+} in urine and live cells; rapid response	[44]
10.	Dansyl-Glu-Pro-Gly-Cys-NH_2	45 nM	Fluorescence "Turn-On" via Photoinduced Electron Transfer (PET)	Sensitive and specific; stable from pH 7–12; applicable to live-cell imaging	[45]
11.	Cbz-APL Peptide-Modified GCE	4.34 fM	Electrochemical via Square Wave Anodic Stripping Voltammetry (SWASV)	Ultra-trace detection; cost-effective; suitable for natural waters	[46]
12.	Pyridine-bis-tyrosine (sMPC)	~11.14 µM	Based on chelation-enhanced fluorescence (CHEF) emission and response	Cost Effective In vivo and In vitro detoxification in Zebra fish and HEK293 cell lines	**Current work**

17. XYZ coordinates for the optimized Geometries with B3LYP-D3/def-SVP:

Py(Tyr)$_2$

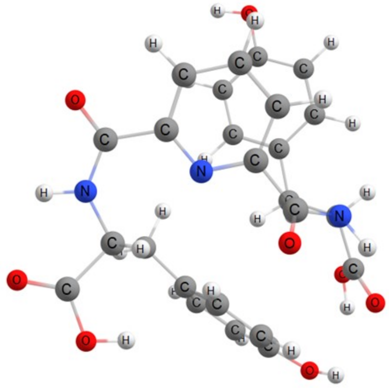

C	-0.687194000	0.698213000	-0.947041000
C	-1.105857000	1.490521000	0.121959000
C	-1.685199000	0.861818000	1.230146000
N	-1.828164000	-0.463202000	1.316209000
C	-1.403093000	-1.227560000	0.305644000
C	-0.838465000	-0.682322000	-0.858206000
H	-0.237739000	1.153915000	-1.831846000
H	-1.000949000	2.575325000	0.104601000
H	-0.536370000	-1.362034000	-1.654517000
C	-1.542274000	-2.741305000	0.346930000
O	-1.243058000	-3.386511000	-0.649868000
C	-2.335113000	1.690306000	2.312114000
O	-3.520225000	1.530000000	2.551235000
N	-2.005421000	-3.364365000	1.467563000
H	-2.140944000	-4.368819000	1.357522000
N	-1.625606000	2.720026000	2.899228000
H	-2.265419000	3.264717000	3.480339000
C	-2.343701000	-2.853691000	2.776924000
H	-2.987335000	-1.971995000	2.665428000

C	-0.301832000	2.697558000	3.524408000
H	0.285705000	3.567017000	3.172224000
C	-3.130732000	-3.943688000	3.512104000
O	-3.356296000	-5.027373000	3.041456000
C	-0.530196000	2.985989000	5.021248000
O	-1.572371000	3.422241000	5.462160000
O	-3.541932000	-3.620676000	4.747239000
H	-3.231466000	-2.734934000	5.005452000
O	0.529631000	2.730291000	5.784564000
H	0.237508000	2.763987000	6.717510000
C	-1.089566000	-2.448639000	3.587552000
H	-0.561677000	-3.355100000	3.926553000
H	-0.417513000	-1.951009000	2.872258000
C	0.549758000	1.433635000	3.327092000
H	1.291581000	1.436393000	4.138350000
H	-0.078145000	0.549404000	3.507423000
C	-1.311109000	-1.499161000	4.744434000
C	-2.314706000	-0.517888000	4.719505000
C	-0.406609000	-1.478633000	5.822065000
C	-2.381478000	0.476364000	5.697658000
C	-0.452083000	-0.482752000	6.794533000
C	-1.426250000	0.522392000	6.717448000
H	-3.030387000	-0.464735000	3.899760000
H	0.375044000	-2.241269000	5.878742000
H	-3.149230000	1.249073000	5.622718000
H	0.277896000	-0.452594000	7.606006000
C	1.293021000	1.285256000	2.016550000
C	1.603782000	-0.000109000	1.553732000
C	1.750025000	2.374258000	1.259891000

C	2.315352000	-0.200306000	0.372229000
C	2.458308000	2.192730000	0.073363000
C	2.738825000	0.898825000	-0.385985000
H	1.266522000	-0.870935000	2.120376000
H	1.541262000	3.396869000	1.583025000
H	2.520955000	-1.217272000	0.024364000
H	2.798976000	3.045084000	-0.517613000
O	-1.371510000	1.548417000	7.616616000
H	-1.986587000	2.243273000	7.328284000
O	3.409278000	0.770222000	-1.559013000
H	3.546240000	-0.166772000	-1.752416000

Cd-Py(Tyr)$_2$

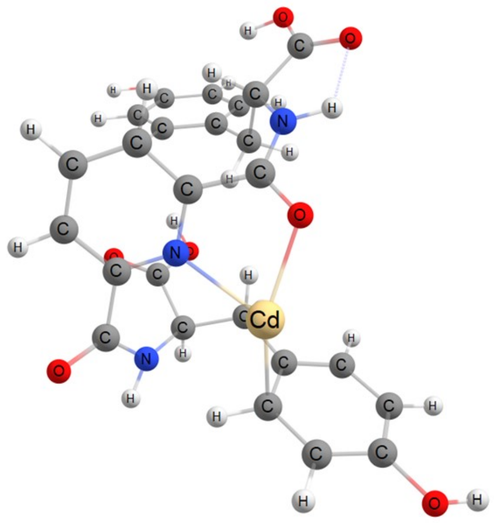

C	-3.927995000	1.355588000	1.306589000
C	-3.053613000	2.433664000	1.340466000
C	-1.681948000	2.200756000	1.151556000
N	-1.213509000	0.975719000	0.886864000
C	-2.050965000	-0.088915000	0.914988000
C	-3.417054000	0.060915000	1.119186000
H	-5.002704000	1.508828000	1.429438000
H	-3.396177000	3.454593000	1.513471000
H	-4.082388000	-0.802590000	1.090367000
C	-1.383280000	-1.407640000	0.641080000
O	-0.465675000	-1.454722000	-0.238162000
C	-0.740419000	3.381323000	1.164342000
O	-1.037931000	4.363226000	0.521916000
N	-1.727611000	-2.473353000	1.335653000
H	-1.320539000	-3.386483000	1.078176000
N	0.459286000	3.291958000	1.846376000
H	0.930091000	4.194927000	1.806565000
C	-2.322438000	-2.502019000	2.678473000
H	-3.330703000	-2.066547000	2.679761000
C	0.860176000	2.453152000	2.971535000

H	1.589783000	3.055133000	3.537301000
C	-2.426523000	-4.002439000	3.029999000
O	-1.925616000	-4.830458000	2.316044000
C	-0.312476000	2.200849000	3.941912000
O	-1.405750000	2.691783000	3.818904000
O	-3.056150000	-4.304471000	4.153951000
H	-3.328240000	-3.520534000	4.663251000
O	0.027351000	1.343939000	4.901918000
H	-0.745282000	1.175068000	5.478548000
C	-1.417553000	-1.736575000	3.690454000
H	-0.494866000	-2.321670000	3.831444000
H	-1.123107000	-0.790237000	3.211073000
C	1.552920000	1.099593000	2.651410000
H	2.051509000	0.777771000	3.575141000
H	0.766616000	0.340210000	2.499470000
C	-2.061496000	-1.419839000	5.015976000
C	-2.881321000	-0.284958000	5.152419000
C	-1.843002000	-2.220182000	6.154430000
C	-3.468019000	0.041661000	6.375904000
C	-2.432305000	-1.912841000	7.376435000
C	-3.256033000	-0.778381000	7.501180000
H	-3.056260000	0.372166000	4.295040000
H	-1.193373000	-3.097245000	6.087657000
H	-4.099081000	0.930895000	6.460710000
H	-2.263035000	-2.533693000	8.258022000
C	2.552651000	0.984501000	1.509946000
C	3.600192000	0.054441000	1.617842000
C	2.442387000	1.668695000	0.249016000
C	4.477001000	-0.214056000	0.564164000

C	3.321419000	1.380327000	-0.834238000
C	4.339121000	0.419962000	-0.687983000
H	3.745575000	-0.477947000	2.561211000
H	1.843059000	2.583528000	0.193315000
H	5.285120000	-0.935943000	0.715845000
H	3.267540000	1.955804000	-1.761955000
O	-3.791401000	-0.536523000	8.703681000
H	-4.357168000	0.248912000	8.695191000
O	5.120834000	0.191708000	-1.738156000
H	5.833578000	-0.439075000	-1.550671000
Cd	0.648898000	0.381893000	-0.354163000

Tyr

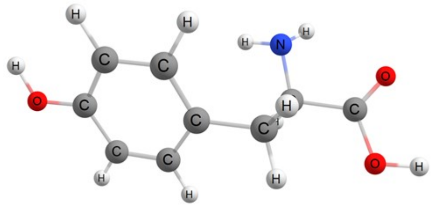

N	-1.290488000	1.610568000	2.895830000
H	-2.223087000	1.907877000	3.180029000
C	-0.291080000	2.437688000	3.543430000
H	-0.142018000	3.428781000	3.054848000
C	-0.783220000	2.790265000	4.939520000
O	-1.925421000	2.679546000	5.313041000

O	0.188140000	3.298842000	5.720448000
H	-0.228864000	3.529177000	6.568567000
C	1.088448000	1.740279000	3.564279000
H	1.800643000	2.384338000	4.099172000
H	0.983109000	0.805426000	4.137041000
C	1.599147000	1.446456000	2.175028000
C	1.211890000	0.286658000	1.487847000
C	2.440284000	2.352333000	1.508079000
C	1.649914000	0.036781000	0.185475000
C	2.886118000	2.116224000	0.208827000
C	2.492339000	0.950301000	-0.462637000
H	0.547133000	-0.426825000	1.979838000
H	2.759766000	3.263634000	2.022524000
H	1.339445000	-0.877249000	-0.331522000
H	3.546377000	2.819550000	-0.302593000
O	2.954319000	0.755989000	-1.726151000
H	2.618750000	-0.084990000	-2.063282000
H	-1.218048000	1.684951000	1.883785000

16. References:

[1] N. Singh, R. Singh, S. Sharma, K. Kesharwani, K. B. Joshi, S. Verma, *New J. Chem.* **2021**, *45*, 153–161.

[2] S. Sharma, A. Kautu, N. Kumar, N. Swain, V. Kumar, R. Singh, K. Kesharwani, N. Singh, P. Gupta, K. Ballabh Joshi, *ChemNanoMat.* **2024**, *10*(10), e202400098.

[3] S. Sharma, A. Kautu, N. Singh, N. Kumar, V. Kumar, R. Singh, K. Kesharwani, N. Swain, P. Gupta, K. B. Joshi, *Next Materials.* **2024**, *4*, 100118.

[4] K. Kesharwani, R. Singh, N. Kumar, N. Singh, P. Gupta, K. B. Joshi, *Nanoscale*. **2022**, *14*, 10200–10210.

[5] A. Kautu, S. Sharma, R. Singh, S. S. Negi, N. Singh, N. Swain, V. Kumar, N. Kumar, P. Gupta, D. Bhatia, K. B. Joshi, *Nanoscale.* **2024**, *16*, 14940–14952.

[6] R. Singh, N. Kumar Mishra, V. Kumar, V. Vinayak, K. Ballabh Joshi, *ChemBioChem.* **2018**, *19*, 1630–1637.

[7] K. Kesharwani, A. Kautu, S. Sharma, R. Singh, V. Kumar, S. K. Tripathi, P. Shukla, K.B. Joshi, *Chem. Commun*. **2022**,*58*, 13815-13818.

[8] F. Neese, *Comput. Mol. Sci.*, **2012**, *2*(1), 73-78.

[9] Neese, *Comput. Mol. Sci*. **2022**, *12*, e1606.

[10] A. D. Becke, *Phys. Rev. A.* **1988**, *38*(6), 3098-3100

[11] J. P. Perdew, *Phys. Rev. B*. **1986**, *33*(12), 8822.

[12] F. Weigend, R. Ahlrichs, *Phys. Chem. Chem. Phys*. **2005**, *7*(18), 3297-3305.

[13] S.Grimme, S. Ehrlich, L. Goerigk, *J. Comput. Chem.* **2011**, *32*(7) 1456-1465.

[14] J. Tomasi, B. Mennucci, R. Cammi, *Chem. Rev.* **2005**, *105*(8), 2999-3094.

[15] B. Das Gupta, A. Halder, T. Vijayakanth, N. Ghosh, R. Konar, O. Mukherjee, E. Gazit, S. Mondal, *J Mater Chem B.* **2024**, *12*, 8444–8453.

[16] H. L. Kewat, Y. N. Chouryal, R. K. Sharma, D. J. Mondal, I. A. Wani, A. Waghmare, S. Konar, S. Nigam, Y. Bhargava, P. Ghosh, *ChemistrySelect.* **2024**, *9*(8), e202303750.

[17] S. Nema, Y. Bhargava, *Zebrafish*. **2017**, *14*(4), 371-378.

[18] H. Gaur, N. Pullaguri, S. Nema, S. Purushothaman, Y. Bhargava, A. Bhargava, *Zebrafish*. **2018**, *15*(3), 254-262.

[19] S. H. Cheng, A. W. K. Wai, C. H. So, R. S. S. Wu, *Environ Toxicol Chem.* **2000**, *19*, 3024–3031.

[20] S. R. Blechinger, J. T. Warren, J. Y. Kuwada, P. H. Krone, *Environ Health Perspect.* e20230375002, *110*, 1041–1046.

[21] H. Wada, *Talanta* **1976**, *23*, 669–671.

[22] S. Sumiyoshi, K. Suyama, D. Tatsubo, N. Tanaka, K. Tomohara, S. Taniguchi, I. Maeda, T. Nose, *Sci Rep.* **2022**, *12*, 1861.

[23] Y. Yeni, H. Nadaroglu, M. S. Ertugrul, A. Hacimuftuoglu, A. Alayli, *Toxicol Rep.* **2024**, *13*, 101818.

[24] K. Shivaji, S. Mani, P. Ponmurugan, C. S. De Castro, M. Lloyd Davies, M. G. Balasubramanian, S. Pitchaimuthu, *ACS Appl Nano Mater.* **2018**, *1*, 1683–1693.

[25] A. Ghasempour, H. Dehghan, M. Ataee, B. Chen, Z. Zhao, M. Sedighi, X. Guo, M.-A. Shahbazi, *Molecules.* **2023**, *28*, 3857.

[26] F. Gerdes, C. Navío, B. H. Juárez, C. Klinke, *Nano Lett.* **2017**, *17*, 4165–4171.

[27] R. Tarrahi, A. Movafeghi, A. Khataee, F. Rezanejad, G. Gohari, *Molecules.* **2019**, *24*, 410.

[28] M. Shakibaie, S. Riahi-Madvar, A. Ameri, P. Amiri-Moghadam, M. Adeli-Sardou, H. Forootanfar, *J Clust Sci.* **2022**, *33*, 1877–1887.

[29] A. Ghasempour, H. Dehghan, M. Ataee, B. Chen, Z. Zhao, M. Sedighi, X. Guo, M.-A. Shahbazi, *Molecules.* **2023**, *28*, 3857.

[30] A. Dixit, R. Bala, B. Pareek, A. Chaudhary, V. S. Jaswal, *Journal of University of Shanghai for Science and Technology.* **2021**, *23*, 462–471.

[31] A. Datta, S. K. Panda, S. Chaudhuri, *J. Phys. Chem. C.* **2007**, *111*, 17260–17264.

[32] F. Gao, Q. Lu, X. Meng, S. Komarneni, *J. Phys. Chem. C.* **2008**, *112*, 13359–13365.

[33] Y. Li, X. Li, C. Yang, Y. Li, *J Mater Chem.* **2003**, *13*, 2641.

[34] H.-B. Kim, D.-J. Jang, *Nanoscale.* **2016**, *8*, 403–410.

[35] G. Jin, Y. Zeng, X. Liu, Q. Wang, J. Wei, F. Liu, H. Li, *Nanomaterials.* **2024**, *14*, 989.

[36] S. S. Warule, N. S. Chaudhari, R. T. Shisode, K. V. Desa, B. B. Kale, M. A. More, *CrystEngComm.* **2015**, *17*, 140–148.

[37] T. C. Pham, Y. K. Kim, J. Bin Park, S. Jeon, J. Ahn, Y. Yim, J. Yoon, S. Lee, *ChemPhotoChem.* **2019**, *3*, 1133–1137.

[38] Shaily, A. Kumar, N. Ahmed, *New J. Chem.* **2017**, *41*, 14746–14753.

[39] N. Yadav, A. K. Singh, *J Electrochem Soc.* **2019**, *166*, B644–B653.

[40] Y. Liu, X. Tang, M. Deng, T. Zhu, L. Edman, J. Wang, *J Alloys Compd.* **2021**, *864*, 158109.

[41] S. Liu, Y. Li, X. Su, *Analytical Methods.* **2012**, *4*, 1365–1370.

[42] C. Wei, X. Wei, Z. Hu, D. Yang, S. Mei, G. Zhang, D. Su, W. Zhang, R. Guo, *Analytical Methods.* **2019**, *11*, 2559–2564.

[43] S. Banerjee, S. Kar, S. Santra, *Chem. Commun.* **2008**, 3037–3039.

[44] K. H. Jung, S. Oh, J. Park, Y. J. Park, S.-H. Park, K.-H. Lee, *New Journal of Chemistry.* **2018**, *42*, 18143–18151.

[45] P. Wang, D. Zhou, B. Chen, *Spectrochim Acta A Mol Biomol Spectrosc.* **2019**, *207*, 276–283.

[46] T. kokab, A. Shah, J. Nisar, A. M. Khan, S. B. Khan, A. H. Shah, *ACS Omega.* **2020**, *5*, 10123–10132.
